# A deep learning method that identifies cellular heterogeneity using nanoscale nuclear features

**DOI:** 10.1038/s42256-024-00883-x

**Published:** 2024-08-27

**Authors:** Davide Carnevali, Limei Zhong, Esther González-Almela, Carlotta Viana, Mikhail Rotkevich, Aiping Wang, Daniel Franco-Barranco, Aitor Gonzalez-Marfil, Maria Victoria Neguembor, Alvaro Castells-Garcia, Ignacio Arganda-Carreras, Maria Pia Cosma

**Affiliations:** 1 Centre for Genomic Regulation (CRG), The Barcelona Institute of Science and Technology, Barcelona, Spain; 2 Medical Research Institute, Guangdong Provincial People’s Hospital (Guangdong Academy of Medical Sciences), Southern Medical University, Guangzhou, China; 3 Department of Computer Science and Artificial Intelligence, University of the Basque Country (UPV/EHU), Paseo Manuel Lardizabal 1, San Sebastian, Spain; 4 Donostia International Physics Center (DIPC), San Sebastian, Spain; 5 Ikerbasque, Basque Foundation for Science, Bilbao, Spain; 6 Biofisika Institute, Barrio Sarrena s/n, Leioa, Spain; 7 ICREA, Barcelona, Spain; 8 Universitat Pompeu Fabra (UPF), Barcelona, Spain

**Keywords:** Single-molecule biophysics, Computational biophysics

## Abstract

Cellular phenotypic heterogeneity is an important hallmark of many biological processes and understanding its origins remains a substantial challenge. This heterogeneity often reflects variations in the chromatin structure, influenced by factors such as viral infections and cancer, which dramatically reshape the cellular landscape. To address the challenge of identifying distinct cell states, we developed artificial intelligence of the nucleus (AINU), a deep learning method that can identify specific nuclear signatures at the nanoscale resolution. AINU can distinguish different cell states based on the spatial arrangement of core histone H3, RNA polymerase II or DNA from super-resolution microscopy images. With only a small number of images as the training data, AINU correctly identifies human somatic cells, human-induced pluripotent stem cells, very early stage infected cells transduced with DNA herpes simplex virus type 1 and even cancer cells after appropriate retraining. Finally, using AI interpretability methods, we find that the RNA polymerase II localizations in the nucleoli aid in distinguishing human-induced pluripotent stem cells from their somatic cells. Overall, AINU coupled with super-resolution microscopy of nuclear structures provides a robust tool for the precise detection of cellular heterogeneity, with considerable potential for advancing diagnostics and therapies in regenerative medicine, virology and cancer biology.

## Abstract (web summary)

Cellular phenotypic heterogeneity is a key determinant of biological functions and is challenging to identify. A deep learning method that recognizes specific nuclear signatures is discussed, which can identify cellular heterogeneity and differentiate between various cell states using a small amount of super-resolution microscopy data.

## Main

Cellular phenotypic heterogeneity is a key determinant of many biological functions; yet, it is still not clear whether it stems from the modifications of the chromatin structure or vice versa. Cell heterogeneity can arise due to external agents that profoundly alter the chromatin structure of the host cells, such as viral infection^1,2^ (consider, for instance, that most of the world adult population is seropositive to herpes simplex virus type 1 (HSV-1) (refs. ^3,4^)). It also arises from chromatin alterations, which are a hallmark of cancer.

Thus, identifying cellular phenotypic heterogeneity can provide key information about biological functions, and revealing the chromatin structure of each cell can be a clear proxy of this heterogeneity. We have previously used single-molecule localization microscopy (SMLM) methods, specifically stochastic optical reconstruction microscopy (STORM), to determine the nanoscale arrangements of chromatin fibres in cells^5^. We found that (1) nucleosomes arrange in groups, called clutches^5^; (2) the density and number of nucleosomes per clutch are the key determinants of the somatic or stem cell state^5,6^; and (3) transcriptionally active clutches are enriched for RNA polymerase II (Pol II) (ref. ^7^). STORM also allowed us to visualize changes in the compaction of the clutch-associated DNA due to the different epigenetic states of somatic and stem cells^8^.

Overall, SMLM represents a major advantage over diffraction-limited imaging, as it permits changes in nuclear nanostructures to be both visualized and quantified at the nanoscale^9^. Current methods of analysing single-molecule spatial distribution, such as cluster algorithms, are powerful at extracting the nuclear locations and their local density. However, it is unclear how the spatial distributions and densities of these molecules can be used for identifying cell states.

With recent advances in artificial intelligence (AI), convolutional neural networks (CNNs) have become the de facto standard for computer vision and a wide range of medical and healthcare imaging applications^10–14^. Deep learning (DL) models have been used to classify whole-cell images and tracking using diffraction-limited microscopy^15–17^. Moreover, super-resolution (SR) microscopy has also been used to enhance localization precision during data acquisition^18,19^ and for semantic segmentation^20,21^ but not yet (to the best of our knowledge) to classify cells based on subcellular structures using SMLM images. Recently developed algorithms can now elucidate and interpret the outputs and decisions of DL models, enabling critical biological features to be identified and—importantly—overcoming the previous limitations of a lack of transparency and interpretability of results^22,23^.

Here we introduce the AI of the nucleus (AINU), a novel method that can effectively train a CNN architecture using minimal training data from nuclear feature imaging. Employing STORM, we imaged Ser 5-phosphorylated RNA Pol II, histone H3 and DNA in diverse cell states, including human somatic cells, human-induced pluripotent stem cells (hiPSCs), human cells infected by HSV-1 and cancer cells. This approach combines CNNs with SMLM data to effectively identify cell heterogeneity and differentiate cell states. Finally, interpretable AI revealed that Pol II localizations within the nucleoli were the key feature recognized by AINU to identify hiPSCs. Overall, AINU emerges as a powerful tool for studying cell heterogeneity through SMLM nuclear imaging, demonstrating strong potential as a diagnostic method.

## Results

### Identification of the optimal architecture for SR imaging

**Fig. 1:**
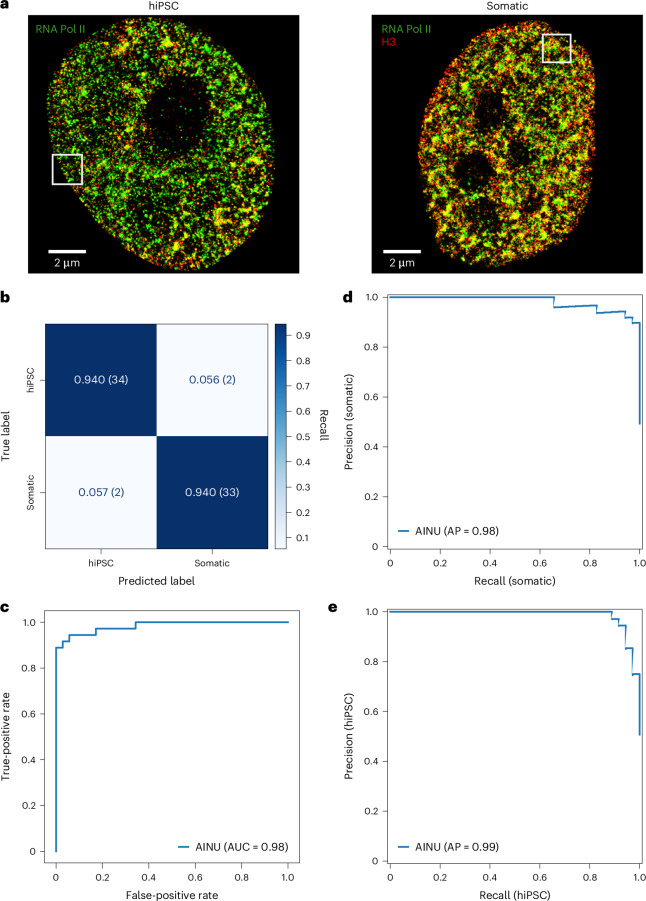
AINU trained with Pol II and H3 images correctly identifies somatic cells and iPSCs. **a**, Representative full-size ×10 rendered images of hiPSCs and somatic cells from dual-colour STORM localizations of Pol II (green) and H3 (red). The white squares include the representative patches reported in Extended Data Fig. 2a. **b**–**e**, AINU trained with images rendered from the dual-colour localizations of Pol II and H3 was challenged on a test set of 71 previously unseen images, representing 20% of the whole dataset. Normalized confusion matrix (with the indication of positively and negatively predicted images in the parentheses) (**b**) shows the performance of the model for each class with the main diagonal showing the accuracy for each class. The ROC curve (**c**) shows the performance of the model at all classification thresholds and the AUC value. **d**,**e**, Precision and recall plots for the somatic (**d**) and hiPSC (**e**) classes reporting the overall average precision (AP).

**Extended Data Fig. 1:**
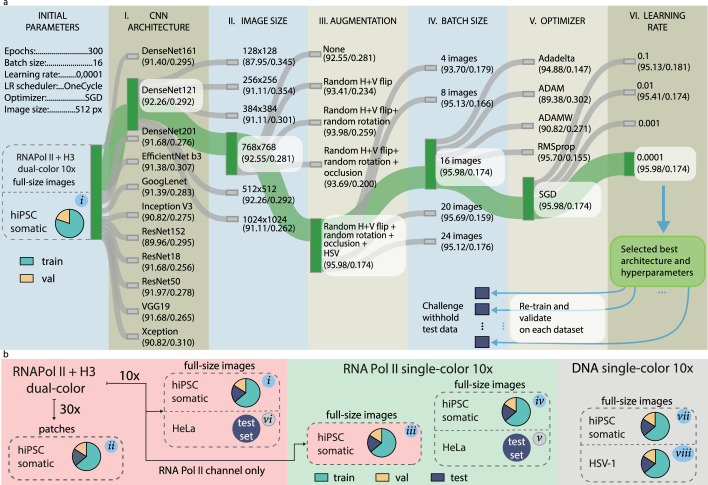
Experimental protocol to find AINU’s optimal configuration. (**a**) Flowchart of our sequential hyperparameter tuning to select the best-performing model architecture using 349 dual-color SR images of Pol II and H3 split in train and validation sets with a 80/20 ratio. The process starts by setting and keeping fixed all the (hyper)parameters and then performing 5-fold CV for 11 different CNN architectures. The best performing one, based on the average accuracy and loss over the 5-folds, is then selected, and the following (hyper)parameter is tested over different values using 5-fold CV. Finally, the best performing architecture and (hyper)parameters are retrained once by randomly splitting the whole dataset in training/validation with a withheld test set, which is used to challenge the trained model using previously unseen images. The green squares connected by arrows highlight the best model or (hyper)parameters resulting from running the 5-fold CV at each step. (**b**) Diagram listing all the different datasets used throughout the paper: (i) dual-color images from 349 full-size nuclei of hiPSC and somatic images: 278 for train/validation and 71 for testing; (ii) image patches rendered from dual-color localization of H3 + RNA Pol II of hiPSC and somatic cells: out of 349 images, 279 were divided into patches for train/validation, and 70 for testing; (iii) 349 full-size single-color images rendered using only the RNA Pol II channel from dual-color localizations; (iv) full-size images rendered from single-color localizations of RNA Pol II of 384 nuclei of hiPSC and somatic cells: 307 nuclei for train/validation, and 77 nuclei for testing; (v) single-color images rendered from RNA Pol II localizations of 30 HeLa cells nuclei; (vi) dual-color images rendered from H3 + RNA Pol II localizations of 33 HeLa cells nuclei; (vii) full-size images rendered from DNA localizations of 185 hiPSC and somatic cells: 148 nuclei for train/validation, and 37 nuclei for testing; (viii) full-size images rendered from DNA localization of 264 A594 cells infected by HSV-1: 211 nuclei for train/validation, and 53 nuclei for testing.

To select the optimal CNN architecture and its hyperparameters for the identification of somatic cells and hiPSCs, we compared 11 distinct CNN architectures based on unique attributes and suitability for our cell classification task, including several models that each offered a different approach and computational efficiency^24^ (Extended Data Fig. 1a). The selection is based on the models’ performance on a total of 349 nuclei dual-colour STORM images of the nucleosome core histone H3 and Pol II (Fig. 1a and Supplementary Table 1). The fluorophores of the selected molecules were collected from human somatic cells and hiPSCs obtained from different somatic cell types and rendered into images having ×10 magnification with respect to the original camera frame (Methods). As a performance measure, we used the average validation accuracy resulting from a stratified five-fold cross-validation (CV) approach^25^, partitioning the dataset with an 80/20 training and validation set ratio (Extended Data Fig. 1a, dataset (i)). For consistency in the comparison, the selection process started by training each of the 11 preselected CNN architectures for 300 epochs and maintaining the same initial hyperparameters, such as the batch size, learning rate, optimizer and image size, without applying data augmentation techniques. Among these architectures, DenseNet-121 (ref. ^26^) was the top performer in identifying somatic cells and hiPSCs, with an average validation accuracy of 92.26 and an average loss of 0.292 (Extended Data Fig. 1a), and was used for subsequent analyses.

Further, to find the most suitable common image size as a network input, we trained and validated the DenseNet-121 model using images rescaled to different pixel sizes. We hypothesized that larger rescaled image sizes would be more accurate due to an improved (for example, closer to original) localization of H3 and Pol II. We found that images resized to 768 × 768 pixels yielded the highest validation accuracy and the lowest validation loss (Extended Data Fig. 1a).

Consequently, we evaluated various data augmentation techniques, batch sizes, optimizers and learning rates (Extended Data Fig. 1a). This outlined workflow enabled us to circumvent the exhaustive process of testing an extensive array of parameter combinations, by, instead, strategically narrowing down our choices at each step. As a result of this procedure, using a batch size of 16 images, the stochastic gradient descent optimizer and a starting learning rate of 0.0001 yielded the highest performance, reaching an average validation accuracy of 95.98 (Extended Data Fig. 1a). In particular, this level of accuracy was achieved despite the relatively limited size of the input dataset, underlining AINU’s ability to accurately identify somatic cells and hiPSCs.

### AINU with dual-colour STORM images identifies hiPSCs

The best architecture was then retrained from scratch using the best training configuration and hyperparameters found in our exhaustive search, without reusing any previous weights. For this retraining phase, we randomly partitioned the whole dataset anew into training, validation and withheld sets, with 20% in the withheld set (Extended Data Fig. 1b, dataset (i)). Training, validation and evaluation on a withheld test set were repeated five times to mitigate any potential biases stemming from random dataset splitting or image heterogeneity. On average, the model achieved a weighted accuracy and F1 score of 0.85 with a standard deviation (s.d.) of 0.07, and an area under the receiver operating characteristic (ROC) (AUC) score of 0.95 ± 0.04 (Supplementary Table 2). In the best dataset split, the model achieved a weighted accuracy and F1 score of 0.94 (Fig. 1b and Supplementary Table 2); an AUC score of 0.98 (Fig. 1c); and average precision and recall scores of 0.98 and 0.99 for somatic cells and hiPSCs, respectively (Fig. 1d,e).

**Extended Data Fig. 2:**
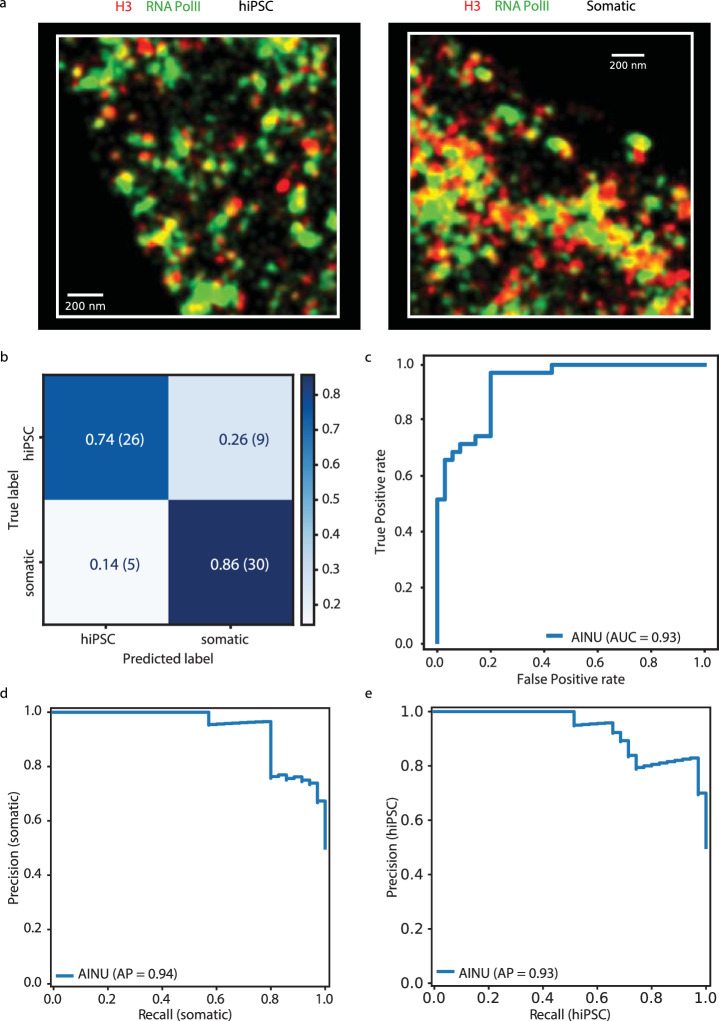
AINU trained with patches and challenged with full-size images. (**a**) Representative patches of 256 × 256-pixel size corresponding to the white square of the image in Fig. 1a rendered at ×30 magnification. (**b–e**) AINU trained with 256 × 256-pixel–wide patches from dual-color images of Pol II and H3 was challenged on a test set composed of patches from 59 previously unseen real images that represented 20% of the whole dataset. (**b**) Confusion matrix reporting normalized and not normalized (in parentheses) values shows the performance of the model for each class with the main diagonals showing respectively the accuracy and the number of positively predicted test samples for each class. The ROC curve (c) shows the performance of the model at all classification thresholds and the value of the AUC. (**d, e**) Precision and recall plots for the somatic cell (d) and hiPSC (e) classes, reporting the overall average precision (AP).

To increase the training and validation datasets and enhance the differentiation between somatic cells and hiPSCs through a high-resolution analysis of nuclear regions, we explored the use of patches extracted from ×30 magnified images (Extended Data Fig. 1b (dataset (ii)) and Extended Data Fig. 2a). This strategy aimed at leveraging localized features by closely examining specific areas of cells. Consequently, we assessed AINU’s performance with this patch dataset to understand the impact of this focused approach. Nevertheless, retraining AINU using patches resulted in a decrease in performance compared with the original training strategy employing full-size images. The model achieved a weighted accuracy and F1 score of 0.80 ± 0.09 (Extended Data Fig. 2b–e and Supplementary Table 3). Note that these results, although very informative, come from different random partitions of the original dataset. Therefore, to validate and compare the results of AINU trained with patches and full-size images, we retrained both models and challenged them on the same test set by extracting patches from the same images that constituted the test set for the full-size image-trained models (Extended Data Fig. 1b, dataset (i); test set only). This methodical approach ensured that both models were evaluated under comparable conditions, using essentially the same data, but presented in different formats (full images versus patches). This way, we confirmed that the model trained on full-size images exhibited superior performance, with a weighted accuracy of 0.85 ± 0.09 (Supplementary Table 4).

**Extended Data Fig. 3:**
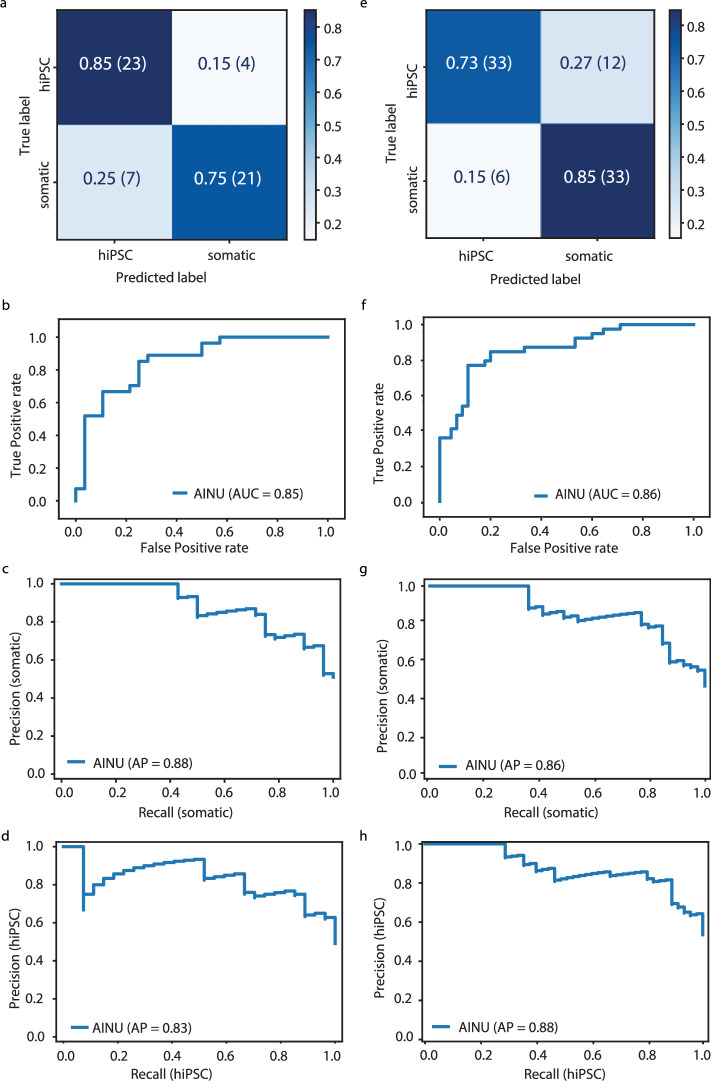
AINU challenged using datasets excluding urine epithelial cells and BJ fibroblasts. (**a-d**) AINU trained with images rendered from dual-color localizations of Pol II and H3 that excluded urine epithelial cells (both somatic and hiPSCs), which were used only as final unseen image test set. (**a**) Confusion matrix reporting normalized and not normalized (in parentheses) values shows the performance of the model for each class with the main diagonals showing respectively the accuracy and the number of positively predicted test samples for each class. The ROC curve (b) shows the performance of the model at all classification thresholds and the value of the AUC. (**c,d**) Precision and recall plots for the somatic cell (c) and hiPSC (d) classes, reporting the overall average precision (AP). (**e-h**) Same as (a–d) for AINU trained with images rendered from dual-color localizations of Pol II and H3 that excluded BJ fibroblasts (both somatic and hiPSCs), which were used only as final unseen image test set.

Next, to test the performance of AINU on unseen images from unseen cell types, we retrained AINU using full-size images (Extended Data Fig. 1b, dataset (i)) but withholding all the images of urine epithelial cells or BJ fibroblasts (each of which contains somatic cells and hiPSCs) for exclusive use as a final test set. AINU still performed well: with a 95% confidence interval, it gave a weighted accuracy and F1 score of 0.80 ± 0.11 when the urine epithelial cells were withheld, and 0.79 ± 0.09 when BJ fibroblasts were withheld (Extended Data Fig. 3a–h and Supplementary Table 5). We then trained the model by withholding different cell types that are present in only one state (either hiPSC or somatic). For instance, when we withheld amniocytes-derived hiPSCs and human mesenchymal stem cells (MSCs), the model still achieved above-average performances, with a weighted accuracy and F1 score of 0.69 ± 0.12 (Supplementary Table 5).

These results suggested that the spatial organization of the imaged molecules was better determined by the cell state (somatic versus hiPSCs) than the cell type, and cell states are better captured when the cell types that are withheld from training and used as a test set belong to the same type.

### AINU trained with Pol II localizations identifies the cell state

**Fig. 2:**
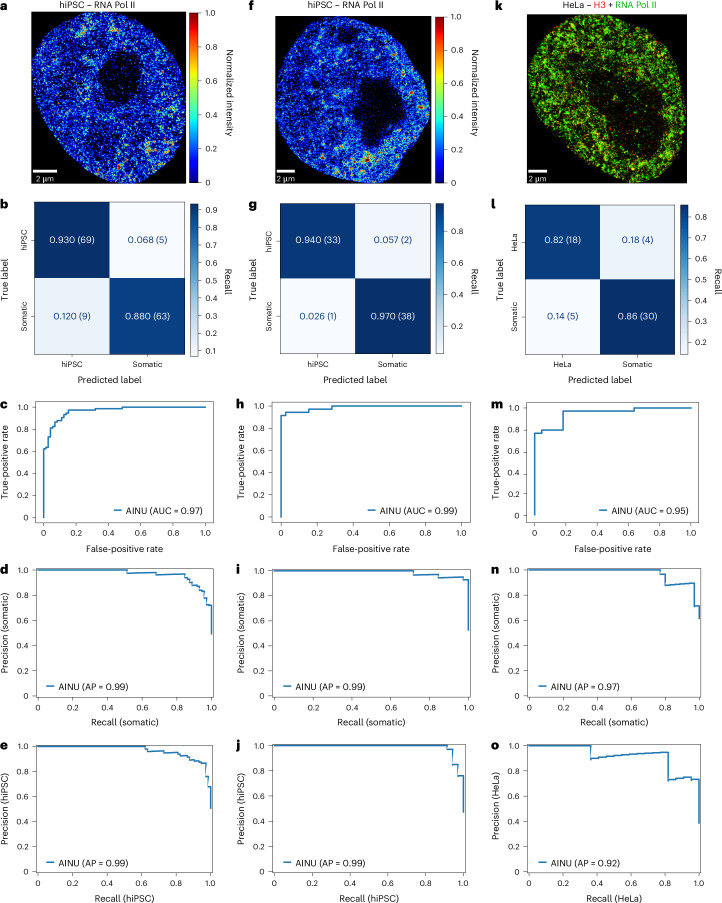
AINU trained with Pol II images correctly identifies somatic cells and iPSCs. **a**, Representative full-size SR image of an hiPSC cell rendered at ×10 magnification using the Pol II channel of dual-colour Pol II and H3 STORM localizations (Methods). The colours correspond to normalized values of the kernel density estimate for each pixel. **b**–**e**, AINU trained with images of Pol II rendered from both dual-colour and single-colour Pol II localizations in hiPSCs and somatic cells was challenged on a test set of 146 previously unseen images, representing 20% of the whole dataset. Normalized confusion matrix (with the indication of positively and negatively predicted images in parentheses) (**b**) shows the performance of the model for each class, with the main diagonal showing the accuracy for each class. The ROC curve (**c**) shows the performance of the model at all classification thresholds and the AUC value. **d**,**e**, Precision and recall plots for the somatic cell (**d**) and hiPSC (**e**) classes, showing the overall AP. **f**, Same data as **a** using Pol II single-colour localizations. **g**–**j**, Same data as **b**–**e** for AINU trained with images from single-colour Pol II localizations and tested on 74 previously unseen images (20% of the whole dataset). **k**, Representative full-size SR image of a HeLa cell rendered at ×10 magnification using the dual-colour localizations of H3 (red) and Pol II (green) (Methods). **l**–**o**, Same data as **b**–**e** for AINU trained with the dual-colour localizations of H3 and Pol II challenged by substituting the hiPSC test images with 33 HeLa images.

As dual-colour STORM imaging is time-consuming and technically challenging, we tested the use of single-colour SR imaging for training. We generated datasets by rendering images from the Pol II channel within the dual-colour SR localizations (Fig. 2a and Extended Data Fig. 1b (dataset (iii))) and added 384 images rendered from single-colour Pol II localizations (Fig. 2f and Extended Data Fig. 1b, dataset (iv)). We conducted five cycles of retraining, validation and testing of AINU, each time using random dataset splitting of these 733 Pol II images. Remarkably, we observed that the model’s performance closely matched that of the model trained with dual-colour images, with an average weighted accuracy of 0.87 ± 0.03 and an average AUC score of 0.95 ± 0.01 (Supplementary Table 6). In the best dataset split, AINU reached an accuracy and F1 score of 0.93 for hiPSCs and 0.88 for somatic cells (Fig. 2b), an AUC score on the withheld set of 0.97 (Fig. 2c) and an average precision and recall of 0.99 for both somatic cells and hiPSCs (Fig. 2d,e).

We then retrained the AINU model for 300 epochs using only the 384 images of single-colour Pol II localizations, repeating this process five times with random data splits. In particular, the model performed slightly better with this smaller dataset, with an average weighted accuracy and F1 score of 0.91 ± 0.05, and an average AUC score of 0.97 ± 0.02 (Supplementary Table 7). Further, it achieved high-performance metrics during the best round of training, validation and splitting (Supplementary Table 7), with 0.94 for hiPSCs and 0.97 for somatic cells (Fig. 2g), an AUC score of 0.99 (Fig. 2h) and average precision and recall of 0.99 for both somatic cells (Fig. 2i) and hiPSCs (Fig. 2j). The increased performance may result from the higher consistency of the single-colour images, all derived from the same experiment, compared with the dual-colour Pol II images. Nonetheless, it is clear that the distribution of Pol II localizations was the primary feature learned by the model, achieving strong performance irrespective of whether dual-colour or single-colour images were used.

### AINU with Pol II localizations identifies cancer cells

Recent studies using STORM have demonstrated that the nanoscale arrangement of chromatin correlates with different stages of carcinogenesis^27^. As this chromatin structure is similar to what we observed in pluripotent cells, we again challenged the AINU model trained with images rendered from RNA Pol II localizations (Extended Data Fig. 1b, dataset (iv)) using the same withheld test set of 39 previously unseen somatic images and replacing the hiPSC images with 33 single-colour, rendered images of Pol II localizations collected in HeLa cells, a type of cancer cell (Fig. 2k and Extended Data Fig. 1b, dataset (v)). We reached a weighted accuracy of approximately 0.78 ± 0.10 (with 95% confidence interval), 0.58 ± 0.17 for HeLa cells and 0.95 ± 0.07 for somatic cells (Supplementary Table 8).

Given that the accuracy in identifying HeLa cells only slightly exceeded (>8%) random chance, we retrained and validated AINU by including six HeLa nuclei images in the training dataset, and two in the validation dataset. The performance of the model with a withheld test set of unseen HeLa images increased notably, reaching a weighted accuracy of 0.84 ± 0.09, with 0.72 ± 0.18 for HeLa cells and 0.92 ± 0.08 for somatic cells (Supplementary Table 8). We next used the same strategy to retrain and validate AINU using dual-colour images of H3 and Pol II and tested on the withheld dataset of dual-colour nuclei images of HeLa cells (Extended Data Fig. 1b, dataset (vi)). The model performances increased further, reaching a weighted accuracy and F1 score of 0.84 ± 0.09 (Fig. 2l and Supplementary Table 8), an AUC score of 0.95 (Fig. 2m) and an average precision and recall of 0.97 for somatic cells (Fig. 2n) and 0.92 for HeLa cells (Fig. 2o). Thus, the AINU model trained with hiPSCs and somatic cells could be used to detect cancer cells after appropriate retraining with a few additional cancer cell images, opening up possible future applications for AINU.

### AINU with SR images of DNA identifies various cell types

**Fig. 3:**
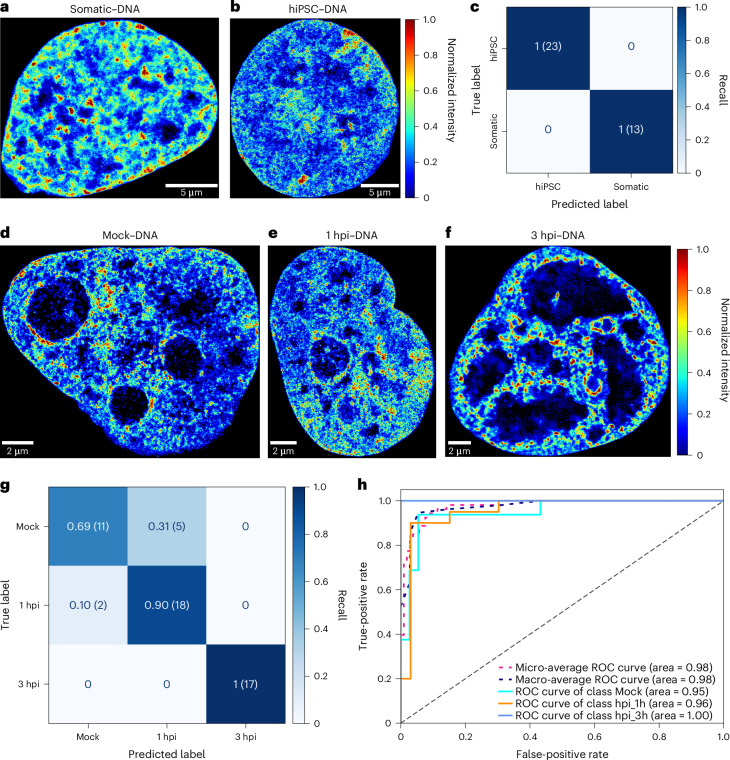
AINU trained with DNA images correctly identifies different cell states. **a**,**b**, Representative full-size SR images of somatic cells (**a**) and hiPSCs (**b**) rendered at ×10 magnification using DNA localizations (Methods). The colours correspond to the values of kernel density estimate for each pixel. **c**, AINU trained with images rendered from single-colour DNA localizations in hiPSCs and somatic cells was challenged on a test set of 36 previously unseen images that represented 20% of the whole dataset. Normalized confusion matrix (with the indication of positively and negatively predicted images in the parentheses) shows the performance of the model for each class, with the main diagonal showing the accuracy for each class. **d**–**f**, Representative full-size SR images of mock-infected (**d**), 1 hpi (**e**) and 3 hpi (**f**) cells rendered using DNA localizations (Methods). The colours correspond to the values of kernel density estimate for each pixel. **g**, Normalized confusion matrix (with the indication of positively and negatively predicted images in the parentheses) shows the performance of the model for each of the three classes, with the main diagonal showing the accuracy for each class. **h**, ROC curves show the performance of the model at all the classification thresholds for each class versus the other two as well as the corresponding value of the AUC. The micro- and macro-average ROC curves of the three classes and their corresponding AUC values are also plotted.

To test whether the same model trained with SR images of DNA could also yield accurate results, we performed SR DNA imaging via click chemistry^8^. We generated a dataset of 185 single-colour SR images of DNA (Extended Data Fig. 1b, dataset (vii)) in human somatic cells (Fig. 3a) and hiPSCs (Fig. 3b), using ten times higher resolution and resizing to 768 × 768 pixels. The model was trained for 300 epochs, with five random splits of the dataset and consistent use of the hyperparameters and augmentation techniques previously used for the full-size single- and dual-colour images. On a withheld test set (Extended Data Fig. 1b, dataset (vii)), the model resulted in an average weighted accuracy of 0.94 ± 0.04 and an average AUC score of 0.98 ± 0.03 (Supplementary Table 9). On the best random split, the model achieved perfect performances, with a weighted F1 score, AUC and accuracy of 1 for both hiPSCs and somatic cells (Supplementary Table 9 and Fig. 3c).

The nuclei of HSV-1-infected cells reorganize to form replication compartments after infection^28–30^. To test the model’s ability to identify early infected cells, lung carcinoma epithelial (A549) cells were mock infected or HSV-1 infected and then imaged at three different time points: mock, 1 hour post-infection (hpi) and 3 hpi. Remodelling of the host chromatin fibres was visually evident as early as 3 hpi (Fig. 3d–f). The model was trained, validated and evaluated on a withheld test set five times with different random splits of the whole dataset (Extended Data Fig. 1b, dataset (viii)). Despite the low number of input images (264), the model performed well on the withheld test set (Extended Data Fig. 1b, dataset (viii)), achieving an average weighted accuracy of 0.83 ± 0.03 and micro- and macro-average AUC scores of 0.95 ± 0.01, over five runs with random splits (Supplementary Table 10). With the best dataset split, the model reached a weighted accuracy and F1 score of 0.87 (Supplementary Table 10), with individual accuracies of 0.69, 0.90 and 1.00 for mock, 1 hpi and 3 hpi cells, respectively (Fig. 3g); and micro- and macro-average AUC scores of 0.98 (Fig. 3h). This suggests that AINU correctly identified HSV-1-infected cells already at 1 hpi, indicating the presence of subtle chromatin remodelling at 1 hpi. We conclude that using DNA SMLM to train a CNN model is a viable option, and that the model can correctly distinguish different levels of chromatin organization.

### Interpretable AI identifies nucleoli as a cell discriminator

We used the Captum framework for PyTorch^31^ to analyse the key features behind the model’s classification and to explain the relationship between the spatial organization of molecules and their impact on biological functions.

**Fig. 4:**
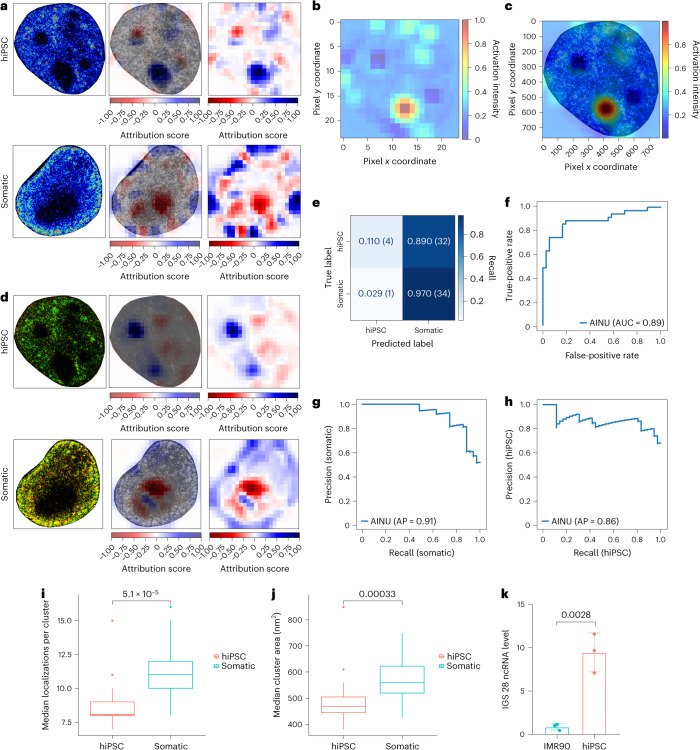
Pol II localizations in the nucleoli are essential for hiPSC identification. **a**, Positive and negative attribution scores for representative hiPSCs and somatic cells using the occlusion-based method (64 × 64 pixel size with stride of 32 pixels) for AINU trained with single-colour Pol II images. The blue regions correspond to areas whose occlusion decreases the probability of predicting the correct class and therefore considered positive for the prediction, whereas the red regions correspond to ‘distracting’ areas whose occlusion increases the probability of predicting the correct class. **b**,**c**, Images showing the CAM data for the hiPSC image in **a** and its overlay on the original image (**c**). **d**, Same data as **a** for AINU trained with dual-colour images of Pol II and H3 in hiPSCs and somatic cells. **e**–**h**, AINU trained with dual-colour Pol II and H3 images was challenged on a test set of 71 previously unseen images having the nucleoli occluded by filling them with cloned non-nucleolous regions from the same image. Normalized confusion matrix (with numbers of positively and negatively predicted images in the parentheses). **e**, Performance of the model for each class; the diagonal reports the accuracy for each class. The ROC curve (**f**) shows the performance of the model at all the classification thresholds and the AUC value. **g**,**h**, Precision and recall plots for the somatic cell (**g**) and hiPSC (**h**) classes, reporting the overall AP. **i**,**j**, Box plots showing a significant difference (two-sided Mann–Whitney *U*-test) between the median localizations per cluster (**i**) and median cluster area (**j**) in somatic (*n* = 22) and hiPSC (*n* = 21) nucleoli. All the box plots depict the median (horizontal line inside box), 25th and 75th percentiles (box), and 25th or 75th percentiles ± 1.5 × interquartile range (whiskers). Distributions were compared as indicated, using the Mann–Whitney *U*-test. **P < 0.01. **k**, Bar plot showing significant difference (two-sided unpaired *t*-test, *P* = 0.0028) of ssRT-qPCR level for asincRNA transcribed by Pol II from rDNA IGS 28, between IMR90 cells and IMR90-derived hiPSCs (error bars represent s.d.). The error bars, including mean and s.d. values, are shown for *n* = 3 independent experiments.

**Extended Data Fig. 4:**
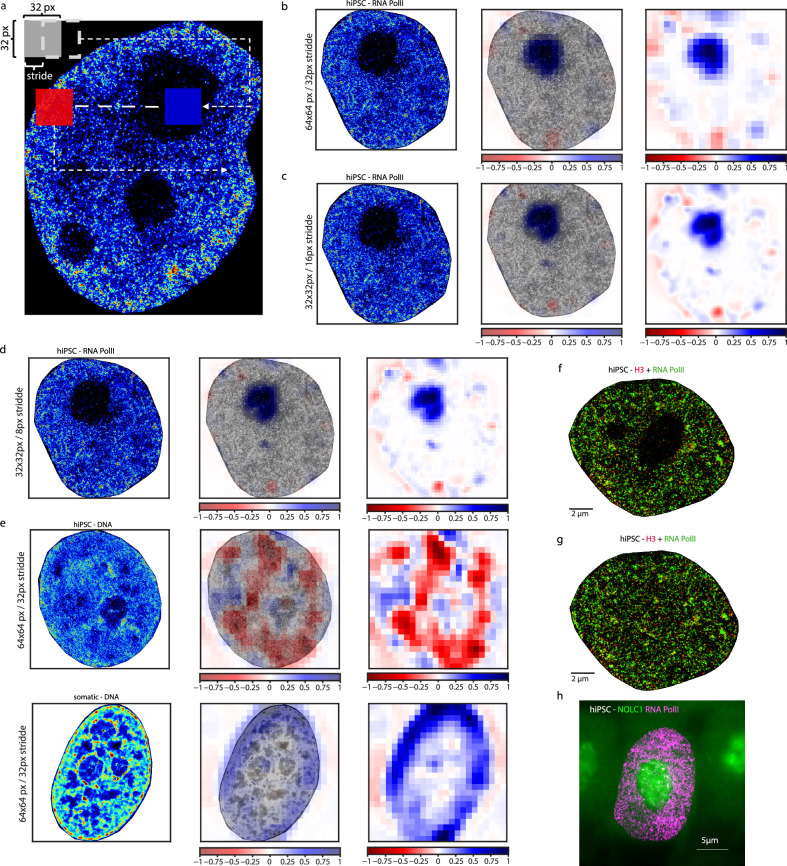
Interpretable AI. (**a**) Occlusion-based method for Interpretable AI. A window of selected size (for example, 32 × 32px) is moved with a specific interval (stride) across the whole image tested by the model in the horizontal and vertical direction. At each occlusion step, the model performs its prediction and compares the probability for the predicted class with that of the non-occluded image. If the probability decreases, a positive attribution score is assigned to the occluded region, whereby the higher the score, the greater the decrease in probability. In contrast, if the probability for the predicted class increases, the occluded region is assigned a negative attribution score, whereby the higher the negative score, the greater the increase of probability. (**b–d**) Positive (blue) and negative (red) attribution scores for one representative hiPSC applying the occlusion-based method, with occlusion region of different sizes and strides as indicated in (a), to AINU trained with single-color Pol II images of hiPSCs and somatic cells. Left, representative super-resolution image of a hiPSC rendered at 10× magnification using single-color Pol II localizations. Middle, overlay of the rendered image with corresponding positive and negative attribution scores. Right, corresponding positive and negative attributions scores only. The blue regions correspond to areas whose occlusion decreases the probability of predicting the correct class, which are considered positive for the prediction, while red regions correspond to ‘distracting’ areas whose occlusion increases the probability of predicting the correct class. (**e**) Same as (b) for AINU trained with single-color DNA images of hiPSCs and somatic cells. (**f**) Representative unmodified super-resolution image rendered at 10× magnification using dual-color Pol II and H3 localizations used to challenge AINU. (**g**) The same as in (f) with the nucleolus replaced with cloned areas from the non-nucleolus corresponding image, to show the decrease of AINU performance for hiPSC identification. (**h**) Representative single-color Pol II (magenta) rendered image of a cell stained with NOLC1 (green) to highlight the nucleolus region.

We first used the occlusion-based algorithm^32^ (Extended Data Fig. 4a) to visually inspect the areas of the image that exhibited positive (contributing) or negative (detracting) attribution scores for each correctly predicted sample, using a 64 × 64 occluding pixel window, with a 32 pixel stride. In particular, the algorithm assigned the highest positive scores (blue) to regions corresponding to nucleoli in hiPSCs, and negative scores (red) to regions at the nucleus’s edge or interior (Fig. 4a, top). Conversely, somatic cells showed the opposite pattern, with positive scores primarily in the nuclear periphery and negative scores within or near the nucleoli (Fig. 4a, bottom).

We then used 32 × 32 pixel occluding windows with different strides (for example, 16 or 8 pixels) for a finer resolution. Consistent with the previous 64 × 64/32 data (Extended Data Fig. 4b), the algorithm still identified the nucleoli as the predominant positive attributions for hiPSCs (Extended Data Fig. 4c,d), with emphasis on specific areas within the nucleoli. These findings were confirmed by applying the class activation mapping (CAM) technique^33^ to each correctly predicted image, which consistently highlighted the nucleoli as the most important feature for hiPSC classification (compare Fig. 4b–c (using CAM) with Fig. 4a (with the occlusion-based method of the same image)).

Using the occlusion-based algorithm and the CAM technique gave consistent results even with H3 and Pol II dual-colour images, with positive values assigned to the nucleoli for hiPSCs and to the outer region of the nucleus for somatic cells (Fig. 4d). Likewise, using the occlusion-based method on images of hiPSCs and somatic cells from the single-colour localization of DNA identified the edge of the nucleus as primarily positive in somatic cells and predominantly negative in hiPSCs (Extended Data Fig. 4e).

To validate the importance of nucleoli for cell-state discrimination, we modified the test set of images by replacing the nucleoli regions with cloned, non-nucleolus areas from the corresponding image (Extended Data Fig. 4f,g). The accuracy of identification was notably decreased for hiPSCs, for which the nucleoli are a crucial distinguishing feature, and slightly increased for somatic cells, in which nucleoli were considered ‘distracting’ areas (for example, with negative attribution scores) (Fig. 4e–h and Supplementary Table 11). These results emphasized the importance of the nucleoli for classifying hiPSCs, and the ability of the model to accurately identify these key structures.

Moreover, we also modified the test set of images by replacing the nucleoli regions with non-nucleolus areas generated using a generative adversarial network technique^34^ and confirmed the same result (Supplementary Information).

Pol II in the nucleolus transcribes antisense intergenic non-coding RNAs (asincRNAs) from rDNA large intergenic spacers (IGSs), thereby controlling RNA polymerase I-derived sincRNAs^35^. Hence, we analysed whether the amount or spatial organization of the Pol II localization signals differed in the nucleoli of 13 hiPSC and 12 somatic cells stained with nucleolar and coiled-body phosphoprotein 1 (NOLC1) (Extended Data Fig. 4h). Our previously developed clustering algorithm^5^ revealed that somatic cells have higher median cluster areas of Pol II, and higher median localizations per cluster in the nucleoli, than hiPSCs, thereby supporting our findings from the occlusion-based and CAM methods (Fig. 4i,j).

Finally, using single-strand reverse-transcription ((ss)RT) quantitative polymerase chain reaction (qPCR) in IMR90-fibroblast somatic cells and IMR90-derived hiPSCs, we observed a significant increase in the expression levels of the asincRNAs transcribed from the rDNA large IGS 28 in hiPSCs compared with somatic cells (Fig. 4k). Overall, these results show that AINU can discriminate hiPSCs from somatic cells by identifying Pol II localizations and activity in the nucleoli of the two cell states.

## Discussion

To analyse cellular phenotypic heterogeneity, we have developed AINU, a CNN model that can correctly identify different cells within a heterogeneous system, even when these cells show only subtle differences in their nuclei that are invisible in diffraction-limited imaging. We achieved this by using the SMLM data collected in multiple cell types labelled for Pol II, core histone H3 or DNA. AINU demonstrated an almost 100% performance in correctly identifying different human somatic cells, hiPSCs and cells infected by HSV-1 at extremely early time points (1 hpi). Importantly, by using interpretable AI algorithms, we discovered that hiPSCs are identified by AINU based on the distinct spatial organization of Pol II in the nucleoli compared with somatic cells.

Large amounts of fluorescence-intensity-based whole-imaged cells are needed as training datasets for DL models^36^. With the use of SR imaging, we can capture nuclear structures, specifically the nanoscale spatial distribution of core histones, Pol II and DNA structure, thereby exploring a novel approach for training DL models. In future research, it will be valuable to investigate the potential of using SMLM data acquired from the imaging of specific epigenetic traits, such as the transcriptionally active or repressive H3Ac, H3K9me3 and H3K27me3 histone marks, as additional nuclear features to train AINU to identify specific cell states. Exploring the combination of multiple nuclear features might lead to enhanced performance in classifying subtle cell-state heterogeneity.

We also trained AINU with SMLM data on stained DNA. Through interpretable AI analysis, we found that AINU correctly discriminates somatic cells from hiPSCs by focusing on the nuclear edge. This may be due to DNA density at the nuclear edge, consistent with previous observations showing compacted heterochromatin at the nuclear periphery in somatic cells^37^. In HSV-1-infected cells, this distinct density might be associated with early replicating viral foci, as the host cells’ chromatin fibre is remodelled. We observed viral replication compartments at 3 hpi that excluded and compacted the host DNA into the non-occupied areas of the nucleus. However, AINU successfully identifies infected cells already at 1 hpi, before any visible appearance of the viral replication compartments. Thus, subtle differences in DNA fibre density at 1 hpi are accurately recognized by AINU at extremely early stages of HSV-1 infection.

Applying interpretable AI, we found that AINU accurately identifies hiPSCs by recognizing the presence of nucleolar Pol II, which leads to an increased transcription of asincRNAs. Pol II in human nucleoli can drive the expression of non-coding intergenic regions flanking nucleolar rRNA genes, thereby generating DNA:RNA hybrid complexes called R-loops and maintaining the nucleolar structure^35^. We, therefore, speculate that specific non-coding RNAs, such as the ones encoded from IGS region 28, are expressed in hiPSCs, where they regulate the functions and maintain cell integrity.

Our results show that AINU, by identifying cellular heterogeneity and ‘understanding’ complex biological systems, presents a potentially robust diagnostic tool with multiple applications. For instance, our method could be used to identify hiPSC clones with high pluripotency grade, using only immunostaining instead of the current requirement for tedious, animal-based experiments. Additionally, our method could be used to identify virus-infected cells in blood or tissue at very early stages after infection, with important applications for immunology and viral biology. Finally (and most importantly, in our opinion), it could identify cancer cells (and perhaps metastatic cells) among wild-type cells from human specimens.

## Methods

### Cells and culture conditions

The following cells were used in this study (all human, except the monkey cells): A549 cells (lung carcinoma, American Type Culture Collection (ATCC) CRL-185); B lymphocytes (GM12878); BJ fibroblasts (human foreskin skin fibroblasts) (ATCC CRL-2522); IMR90 (fibroblasts isolated from lung tissue, ATCC CCL-186); bone marrow MSCs; HeLa; Müller glia; myocardiocytes; spontaneously arising retinal pigment epithelium (ARPE-19); urine epithelial cells; and Vero (African green monkey) cells. Further, hiPSCs were induced from amniocytes, BJ fibroblasts, dermal fibroblasts, normal lung tissue fibroblasts (hiPS(IMR90)-4: WiCell, #WISCi004) periosteum cells, umbilical cord MSCs and urine epithelial cells. HeLa, BJ fibroblasts, IMR90 fibroblasts and myocardiocytes were cultured in Dulbecco’s modified Eagle’s medium (DMEM) (Gibco, #C11995500BT) supplemented with 10% fetal bovine serum (FBS) (Gibco, #10099141) and 1% penicillin–streptomycin (pen/strep) (Gibco, #15140122). Urine epithelial cells were cultured in Lonza REGM BulletKit (Lonza, #CC-3190) supplemented with 1% pen/strep. B lymphocytes were cultured in RPMI 1640 with l-glutamine (Corning Cellgro, #10-040-CVR) supplemented with 15% FBS and 1% pen/strep. Müller glia and ARPE-19 cells were cultured in DMEM/F-12, GlutaMAX (Gibco, #10565018) supplemented with 10% FBS and 1% pen/strep. Bone marrow MSCs were cultured in DMEM supplemented with 0.01% human FGF-basic (Peprotech, #100-18B), 10.00% FBS and 1.00% pen/strep. All the hiPSCs were cultured in mTeSR1 (Stem Cell, #85850) supplemented with 1% pen/strep. A549 cells were cultured in F-12K (Kaighn’s) medium (Gibco, Thermo Fisher Scientific, #21127022) supplemented with 10% FBS (Thermo Fisher Scientific, #10270106) and 1% pen/strep. Vero cells were culture in DMEM, high-glucose GlutaMAX Supplement (Thermo Fisher Scientific, #10566016) supplemented with 5% FBS and 1% pen/strep.

### HSV-1 sample preparation and cell infection

HSV-1 (KOS strain) was grown in Vero cells. Virus preparations were titrated by a plaque assay, as previously reported^38^. For imaging purposes, A549 cells were plated in borosilicate-glass-bottom eight-well chambers (μSlide ibidi, #80827) at a confluency of 20,000–30,000 cells cm^–2^. A549 cells were cultured with media supplemented with 5 µM ethynil-deoxy-cytdine (EdC) (Sigma-Aldrich, #T511307) for 64 h before HSV-1 infection. After media wash, cells were mock infected or infected with HSV-1 at multiplicity of infection of 3 for 1 h at 37 °C in a humidified incubator with 5% CO_2_. The medium was then removed and replaced by fresh DMEM medium (supplemented with 2% FBS). At 1 or 3 hpi, cells were fixed with 4% paraformaldehyde (Alfa Aesar, #43368) for 15 min at room temperature and then rinsed with PBS three times for 5 min each.

### Immunolabelling for SR imaging (STORM)

Cells were plated in chambered coverglass (µ-Slide 8 Well Chamber Slide, ibidi, #80826) at a concentration of 40,000 cells cm^–2^ in growth medium for 24 h. Cells were fixed with 4% paraformaldehyde (Leagene, #DF10315) for 15 min at room temperature and then rinsed with PBS three times for 5 min each. Cells were permeabilized with 0.4% (v/v) Triton X-100 (Acros Organics, 327371000) in PBS for 15 min and then blocked in blocking buffer (10.00% bovine serum albumin, #B2064-50G; 0.01% (v/v) Triton X-100 in PBS) for 1 h at room temperature. Cells were incubated with primary antibodies (anti-RNA polymerase II CTD repeat YSPTSPS (phospho S5) antibody, Abcam, #ab5131; Histone H3 antibody, Active Motif, #39763) in blocking buffer at 1:50 dilution for STORM of both single- and dual-colour imaging, overnight at 4 °C. On the second day, cells were washed three times for 5 min each with wash buffer (2.00% bovine serum albumin and 0.01% Triton X-100 in PBS) and incubated in secondary antibody. For single-colour imaging of Pol II, the secondary antibody (donkey anti-rabbit IgG H&L (Alexa Fluor 647), Abcam, #ab150075) was added at 1:200 dilution in blocking buffer for 1 h at room temperature. For dual-colour imaging, home-made^39^ dye pair (Alexa Fluor 405 NHS Ester, Thermo Fisher, #A30000; Cy3 Maleimide Mono-Reactive Dye Pack, Sigma-Aldrich, #GEPA23031; Alexa Fluor 647 NHS Ester (succinimidyl ester), Thermo Scientific, #A20006) was used to label secondary antibodies (AffiniPure donkey anti-rabbit IgG (H + L), Jackson ImmunoResearch, #711005152; Peroxidase AffiniPure goat anti-mouse IgG, Fcγ subclass 1 specific, Jackson ImmunoResearch, #115-005-205), which were added at a 1:50 dilution in blocking buffer and were incubated for 1 h at room temperature. Cells were then washed three times for 5 min each with wash buffer, and once with PBS. Samples were stored in PBS at 4 °C before imaging.

For Extended Data Fig. 4h, anti-RNA polymerase II CTD repeat YSPTSPS (phospho S5; Abcam #ab5408) was used for STORM and anti-NOLC1 (Abcam #ab184550) was used to mark the nucleoli (both diluted 1:50). Secondary antibodies (goat anti-mouse IgG Alexa Fluor 647, Thermo Scientific #A21235; and goat anti-rabbit IgG, Oregon Green 488, Thermo Scientific #O-11038) were used at 1:250 dilution. Immunostaining was performed using the protocol given above. The complete STORM protocol can be found in another work^40^.

### STORM imaging

STORM imaging was performed as follows: (1) for proteins, using an N-STORM microscope (Nikon) equipped with a CFI SR HP Apochromat TIRF 100× AC oil objective and an ORCA-Flash4.0 V3 digital CMOS camera; (2) for DNA, using Oxford Nanoimager (ONI); and (3) for Pol II and conventional for NOLC1 (Extended Data Fig. 4h), using an N-STORM 4.0 microscope (Nikon) with an iXon Ultra 897 camera (Andor) and a CFI HP Apochromat TIRF ×100 1.49 oil objective, under the highly inclined and laminated optical sheet illumination mode. NIS element (4.60 and 5.21) software was used for N-STORM image acquisition. Only nuclei that were morphologically coherent with the characteristics expected of an interphase nucleus were imaged.

For single-colour STORM imaging of Pol II, continuous imaging acquisition was performed with simultaneous 405 and 647 nm illumination of the sample and 10 ms exposure time for 60,000 frames. The 647 nm laser was used at constant 3.5 kW cm^–2^, and the 405 nm laser power was gradually increased over the imaging period.

For the dual-colour STORM imaging of Pol II and H3, a double activator and single reporter strategy was used by combining AF405_AF647 anti-mouse and Cy3_AF647 anti-rabbit secondary antibodies. Sequential imaging acquisition was performed (one frame of 405 nm activation, followed by four frames of 647 nm reporter; and one frame of 560 nm activation, followed by four frames of 647 nm reporter) with 20 ms exposure time for 120,000 frames. The 647 nm laser was used at a constant 3.5 kW cm^–2^ power density, whereas the powers of the 405 and 560 nm lasers were gradually increased over the imaging period.

Single-colour imaging of DNA images was performed on the Nanoimager S Mark II from ONI equipped with an Olympus 1.4-numerical-aperture ×100 oil-immersion super apochromatic objective and a Hamamatsu sCMOS Orca-Flash4.0 V3. Continuous imaging acquisition was performed with simultaneous 647 nm illumination of the sample, 10 ms exposure time for 45,000 frames.

During SR imaging, freshly prepared buffer was added to the sample: 100 mM cysteamine MEA (Sigma-Aldrich, #30070), 1% Glox solution (0.5 mg ml^–1^ glucose oxidase, 40 mg ml^–1^ catalase; Sigma-Aldrich, #G2133 and #C100), 5% glucose (Sigma-Aldrich, #G8270) in PBS. The buffer was exchanged every hour to avoid enzymatic activity decay and maximize the signal-to-noise ratio of the localizations.

For the ONI images, localization lists were obtained using the integrated simultaneous localization software from ONI (ONI NimOS v. 10.5) and subsequently converted into files compatible with Insight3 (ref. ^41^) for post-processing using custom-built software in MATLAB 2016a.

N-STORM and ONI microscopes have a resolution of 160 and 117 nm pixel^–1^, respectively. For ONI images, molecule localizations were extracted and exported with ONI NimOS v.10.5 and then converted into insight3-compatible files for post-processing with MATLAB 2016a. For N-STORM images, molecule localizations were extracted using Insight3 (ref. ^41^), as described earlier^39,42^. Specifically, point spread functions (PSFs) from the emission from single fluorophores were identified at individual frames of the acquired videos, based on restrictive set thresholds of intensity, PSF size and symmetry. They were fit to a two-dimensional Gaussian from which the centroid of the PSF was obtained and the *x* and *y* positions for 2D STORM imaging were then obtained from the PSF.

All the images were acquired using HiLo^43^ to maximize the signal-to-noise ratio in the nucleus. Focus plane was set to *z* of the maximum nucleus area to ensure the representativity of slices. Cells with uneven intensity profiles or localization artefacts were manually discarded before proceeding with the DL pipeline.

### EdC incorporation and DNA labelling

To label DNA, a 48 h incorporation of 5 µM EdC (5-ethynyl-2′-deoxycytidine; Sigma-Aldrich, #T511307) was performed using the following cells: HeLa, BJ fibroblast, myocardiocytes, urine epithelial cells, B lymphocytes, Müller glia, ARPE-19, MSCs and hiPSCs induced from amniocytes, BJ fibroblasts, dermal fibroblasts, periosteum cells, umbilical cord MSCs and urine epithelial cells. Cells were plated in chambered coverglass (µ-Slide 8 Well Chamber Slide, ibidi, #80826) at a concentration of 40,000 cells cm^–2^ in growth medium supplemented with 5 µM EdC for 24 h. For somatic cells, the growth medium was replaced on the second day with resting medium supplemented with EdC and incubated for another 24 h. For hiPSCs, 10 µM ROCK Inhibitor (Y-27632, Millipore, SCM075) was added together with 5 mM EdC for the first 24 h and then was replaced with medium supplemented with 5 mM EdC only. Cells were fixed with 4% paraformaldehyde (Leagene, #DF10315) for 15 min at room temperature and then rinsed with PBS three times for 5 min each. Cells were permeabilized with 0.4% Triton X-100 in PBS for 15 min and rinsed with PBS three times for 5 min each. The click chemistry reaction was performed by incubating cells for 45 min at room temperature in click chemistry buffer (150 mM HEPES pH 8.2 (Sigma-Aldrich, #7365-45-9), 50 mM aminoguanidine (Sigma-Aldrich, #396494), 100 mM l-ascorbic acid (Sigma-Aldrich, #A92902), 1 mM CuSO_4_ (Sigma-Aldrich, #C1297), 2.0% glucose (Sigma-Aldrich, #G8270), 0.1% Glox solution (see the ‘STORM imaging’ section) and 10 mM AF647 azide (Thermo Fisher Scientific, #A10277)), protected from light. After three washes with PBS, STORM imaging was directly carried out for single-colour DNA imaging experiments (see the ‘STORM imaging’ section).

### (ss)RT-qPCR amplification of asincRNAs

#### RNA extraction

hiPSCs (generated from IMR90, WiCell, no. WISCi004) and IMR90 cells at 70–80% confluency were washed with RNase-free PBS before RNA isolation using a Qiagen RNeasy Mini Kit (Qiagen, #74106). The isolated RNA was treated with DNase I enzyme (Thermo Fisher Scientific, #18068015) and precipitated with 0.8 M trisodium citrate and 1.2 M NaCl.

#### Strand-specific complementary DNA synthesis

IGS region 28 was chosen based on its identification as one of the regions prominently transcribed by Pol II in the nucleolus^35^. The complementary DNA synthesis reaction was set up for the transcript of interest using M-MLV reverse transcriptase (Thermo Fisher Scientific, #28025013). For this reaction, we used the following strand-specific primers in concentrations of 10 µM:

**Table:** 

Primer	Sequence (5′ to 3′)	Company
ssTag_hIGS-28 FS-A	CGAGGATCATGGTGGCGAATAACCTTCCACGAGAGTGAGAAG	Integrated DNA Technologies
7SK FS-A	TAATACGACTCACTATAGGGAGGACCGGTCTTCGGTCAA	Integrated DNA Technologies

The segments were amplified for 10 min at 25 °C, 60 min at 37 °C and 25 min at 70 °C. Samples were diluted 1:10.

#### qPCR

Real-time qPCR was performed using a ViiA 7 Real-Time PCR System (Thermo Fisher Scientific). The qPCR reactions (10 µl total) each contained LightCycler 480 SYBR Green I Master (Roche Diagnostics, #04887352001), 10 µM of each of the forward (strand-specific) and reverse (ssTag) primers, and 2 ng of diluted complementary DNA, as detailed below:

**Table:** 

Primer	Sequence (5′ to 3′)	Company
IGS 28 R	GACCTCCCGAAATCGTACAC	Integrated DNA Technologies
7SK RNA R	TCATTTGGATGTGTCTGCAGTCT	Integrated DNA Technologies
7SK-Tag	TAATACGACTCACTATAGGG	Integrated DNA Technologies
ssTag	CGAGGATCATGGTGGCGAATAA	Integrated DNA Technologies

Amplification of the 7SK region was used for normalization. PCR reactions used the following parameters: 1 cycle of 95 °C for 5 min; 45 cycles of 95 °C for 10 s, 58 °C for 10 s and 72 °C for 19 s; and a final melting curve cycle of 60 °C to 97 °C in 0.05 °C s^–1^ steps. The quantification of each IGS was normalized for the 7SK region.

#### Hardware and software

All the computational operations were conducted using the Center for Genomic Regulation cluster infrastructure, which comprises ten NVIDIA GeForce 2080 Ti with 11 GB of memory per GPU. The DL computations were performed using the PyTorch framework version 1.10, with CUDA drivers version 11.3 and Python version 3.9.7. For ONI images, molecule localizations were extracted and exported with ONI NimOS v. 10.5 and then converted into Insight3-compatible files for post-processing with MATLAB 2016a. For N-STORM images, molecule localizations were extracted using Insight3. The images were segmented by manually selecting the nuclear areas corresponding to each cell nucleus with Fiji^44^. Plots and statistical tests shown in Fig. 4i–k were produced with R version 4.2.2 and GraphPad Prism version 8.01.

#### Datasets

We collected a total of 349 dual-colour images, including 171 from human somatic cell nuclei and 178 from hiPSC nuclei, featuring H3 and Pol II. Additionally, 384 single-colour images of Pol II were obtained, with 187 from somatic cells and 197 from hiPSCs. Somatic cells comprised different cell types (B lymphocytes, bone marrow MSCs, Müller glia, myocardiocytes, BJ fibroblasts, ARPE-19 and urine epithelial cells) and hiPSCs were generated from some of the somatic cells included in the study (Supplementary Table 1).

We also collected single-colour images of Pol II (33) and dual-colour images of H3 and of Pol II (30) in the nuclei of HeLa cells, single-colour images of DNA in human somatic cells (68), hiPSCs (117) as well as images of DNA in nuclei of mock-infected (81) and HSV-1-infected A549 cells at 1 hpi (98) and 3 hpi (85). To avoid sex chromosome bias during model training, we included images from both male and female cells.

The molecule localization coordinates of the selected region of interest (that is, nucleus) of dual-colour Pol II and H3 were multiplied either by 10 or 30 corresponding to a resolution of 16.0 and 5.3 nm pixel^–1^, respectively, and then rendered into images by computing a Gaussian kernel density estimate using Scikit-learn^45^ KDTree function. The images were finally resized with the desired dimensions to be used as an input of the CNN and the aspect ratio was maintained.

To enable the model to differentiate between the background of the image outside the nucleus and regions within the nucleus lacking localizations, we set the background of full-size ×10 rendered images to white.

Both single- and dual-colour images rendered at ×10 magnification (Fig. 1a) were directly used as train/validation and test sets (see the ‘Workflow for identifying the best-performing model’ section), whereas dual-colour ones rendered at ×30 magnification were divided in non-overlapping patches (Extended Data Fig. 2a) of three different sizes (256 × 256, 512 × 512 and 768 × 768 pixels). Only patches with more than 75% of the pixels containing localizations were used as training/validation and test sets.

To avoid changing the aspect ratio, which would have modified the spatial relationship of the imaged molecules, each image was first resized on its longest axis to the desired value (for example, 768 pixels for full-size images) by keeping the aspect ratio and was subsequently padded with the background colour on the shortest axis. For all the other datasets (that is, single-colour Pol II, DNA and dual-colour Pol II and H3 HeLa), we just rendered and used images from ×10 magnification of the molecule localizations.

#### Workflow for identifying the best-performing model

The dual-colour dataset, consisting of 349 full-size images rendered at ×10 magnification, underwent an 80/20 split into training and validation sets, keeping the proportion of each cell class present in the dataset. Employing a stratified *K*-fold CV (with five folds)^25^, we explored 11 different CNN architectures, each trained for 300 epochs using images resized to 512 × 512 pixels. Model hyperparameters remained constant across architectures for fair comparisons (Extended Data Fig. 1a). The best-performing architecture was chosen based on the average accuracy and loss over the five folds. Data augmentation techniques, including flipping, random rotation and colour adjustments, were applied using the Albumentations library^46^. Region dropout and the GridMask technique^47^ were explored to enhance training data diversity (as shown in Extended Data Fig. 1a). The use of the GridMask technique with a ratio of mask holes to unit size set at 0.3 and grid unit sizes ranging from 128 to 384 led to the highest average accuracy and lowest average loss values when assessed through five-fold CV (Extended Data Fig. 1a).

Systematically, we reset the model weights with the best-performing parameters based on the validation set during the training stage. The final architecture with its optimal hyperparameters, named AINU, was used for training, validation and evaluation across various datasets, including single-colour images of Pol II localizations collected in somatic cells and hiPSCs; dual-colour images of Pol II and H3 collected from HeLa cells; and single-colour images of DNA collected from somatic cells, hiPSCs and HSV-1-infected A549 cells.

To explore alternative training approaches, we employed a patch dataset, using patches extracted from the dual-colour STORM images rendered at higher resolutions such as ×30 instead of ×10 (Extended Data Fig. 1b, dataset (ii)). This approach was undertaken to increase the training dataset and to uncover potentially hidden nuclear features that could improve the model performance. To collect patches, the localization coordinates of each blinking fluorophore were multiplied by a factor of 30, effectively rendering images at a ×30 magnification relative to the original camera pixel size. These high-resolution images were then subdivided into smaller, non-overlapping patches, of 256 × 256 or 512 × 512 pixels, effectively increasing the size of the dataset.

The performance of the AINU model trained with patches was evaluated using a separate withheld test set, selected randomly and thus different from those used with full-size images (Extended Data Fig. 1b, grey sector in dataset (ii)). For evaluation, we used various metrics, including the F1 score, validation accuracy and loss, AUC, precision and recall^48,49^. Due to the intrinsic differences between the full-size dual-colour images and patch-based images, fine-tuning of AINU was needed when training with patches or simulated images.

When evaluating the model trained with patches, a classification threshold of 50% was applied as the minimum number of patches correctly assigned to the positive class. This means that an image was correctly predicted if more than 50% of its patches were assigned to the true class.

No transformations that altered the aspect ratio of the image were applied to augment the data, as this would have resulted in a distorted spatial relationship of the imaged molecules, which is a key characteristic feature of somatic and hiPSCs.

Because those models were not trained five times with random dataset splits due to high resource consumption, the performance scores were reported with the 95% confidence interval calculated with the normal approximation method.

When training was performed using patches, AINU exhibited optimal validation performance when using non-overlapping patches of 256 × 256 pixels. However, when challenged with patches from the test set of full-size images (Extended Data Fig. 1b, dataset (i)), it showed lower performance than the original full-size image training strategy (see the ‘Results’ section). This suggests that using only partial sections of the nucleus, even if at higher magnification, does not improve the model’s overall performance.

#### Interpretable AI and cluster analysis

To evaluate the contribution of each input feature (image pixels) on the model’s output and prediction accuracy and to gain an understanding of the model’s decisions, the Captum framework^31^ and its occlusion-based method were used. This approach allowed to visually inspect the areas of the image that exhibited positive or negative attribution scores for each correctly predicted sample (Extended Data Fig. 4a). Positive attribution scores indicate areas of the image that contribute to the model’s prediction, whereas negative attribution scores highlight regions whose removal increases the probability of the predicted class. To apply the occlusion technique, we systematically slid a 64 × 64 pixel window, with a 32 pixel stride, across each image generated from the Pol II localizations. Subsequently, we superimposed the heat map of attribution scores onto the original image, facilitating the identification of the image regions most influential in the model’s classification decision.

These results were further validated using the CAM^33^ method, which highlighted specific regions within the nucleoli that are critical for classification. Additionally, the differences in localization quantification, distribution and organization inside the nucleoli of hiPSCs and somatic cells were verified using a distance-based clustering algorithm, as detailed elsewhere^5^ (Fig. 4i,j).

### Reporting summary

Further information on research design is available in the Nature Portfolio Reporting Summary linked to this article.

## Supplementary information

Supplementary Information Supplementary Figs. 1 and 2.

Reporting Summary

Supplementary Tables 1–11 Supplementary Tables 1–11 categorize the types and quantities of images used in this study and provide detailed performance statistics for the various models tested, including metrics such as accuracy, precision, recall and F1 score.

## Data Availability

The trained model and the full dataset for dual-colour images generated during the current study are available via Code Ocean at 10.24433/CO.7405455.v2 (ref. ^50^). For single-colour images (Pol II and DNA), only the trained models and test sets are available to reproduce the results of the paper.

## Code availability

All the codes used to process and analyse the data and to train, validate and test the AINU are available via Code Ocean at 10.24433/CO.7405455.v2 (ref. ^50^).

## References

[1] Aho, V. et al. Chromatin organization regulates viral egress dynamics. *Sci. Rep.* **7**, 3692 (2017). 28623258 10.1038/s41598-017-03630-y PMC5473834

[2] Aho, V. et al. Infection-induced chromatin modifications facilitate translocation of herpes simplex virus capsids to the inner nuclear membrane. *PLoS Pathog.* **17**, e1010132 (2021). 34910768 10.1371/journal.ppat.1010132 PMC8673650

[3] Looker, K. J. et al. Global and regional estimates of prevalent and incident herpes simplex virus type 1 infections in 2012. *PLoS ONE* **10**, e0140765 (2015). 26510007 10.1371/journal.pone.0140765 PMC4624804

[4] James, C. et al. Herpes simplex virus: global infection prevalence and incidence estimates, 2016. *Bull. World Health Organ.* **98**, 315–329 (2020). 32514197 10.2471/BLT.19.237149 PMC7265941

[5] Ricci, M. A., Manzo, C., García-Parajo, M. F., Lakadamyali, M. & Cosma, M. P. Chromatin fibers are formed by heterogeneous groups of nucleosomes in vivo. *Cell* **160**, 1145–1158 (2015). 25768910 10.1016/j.cell.2015.01.054

[6] Gómez-García, P. A. et al. Mesoscale modeling and single-nucleosome tracking reveal remodeling of clutch folding and dynamics in stem cell differentiation. *Cell Rep.* **34**, 108614 (2021). 33440158 10.1016/j.celrep.2020.108614 PMC7842188

[7] Castells-Garcia, A. et al. Super resolution microscopy reveals how elongating RNA polymerase II and nascent RNA interact with nucleosome clutches. *Nucleic Acids Res.* **50**, 175–190 (2022). 34929735 10.1093/nar/gkab1215 PMC8754629

[8] Otterstrom, J. et al. Super-resolution microscopy reveals how histone tail acetylation affects DNA compaction within nucleosomes in vivo. *Nucleic Acids Res.* **47**, 8470–8484 (2019). 31287868 10.1093/nar/gkz593 PMC6895258

[9] Lakadamyali, M. & Cosma, M. P. Visualizing the genome in high resolution challenges our textbook understanding. *Nat. Methods* **17**, 371–379 (2020). 32123395 10.1038/s41592-020-0758-3

[10] Esteva, A. et al. Dermatologist-level classification of skin cancer with deep neural networks. *Nature* **542**, 115–118 (2017). 28117445 10.1038/nature21056 PMC8382232

[11] Kamnitsas, K. et al. Efficient multi-scale 3D CNN with fully connected CRF for accurate brain lesion segmentation. *Med. Image Anal.* **36**, 61–78 (2017). 27865153 10.1016/j.media.2016.10.004

[12] De Fauw, J. et al. Clinically applicable deep learning for diagnosis and referral in retinal disease. *Nat. Med.* **24**, 1342–1350 (2018). 30104768 10.1038/s41591-018-0107-6

[13] Lee, J. H. & Kim, K. G. Applying deep learning in medical images: the case of bone age estimation. *Healthc. Inform. Res.* **24**, 86–92 (2018). 29503757 10.4258/hir.2018.24.1.86 PMC5820091

[14] Sato, M. et al. Application of deep learning to the classification of images from colposcopy. *Oncol. Lett.* **15**, 3518–3523 (2018). 29456725 10.3892/ol.2018.7762 PMC5795879

[15] Pratapa, A., Doron, M. & Caicedo, J. C. Image-based cell phenotyping with deep learning. *Curr. Opin. Chem. Biol.* **65**, 9–17 (2021). 34023800 10.1016/j.cbpa.2021.04.001

[16] Maška, M. et al. The cell tracking challenge: 10 years of objective benchmarking. *Nat. Methods* **20**, 1010–1020 (2023). 37202537 10.1038/s41592-023-01879-y PMC10333123

[17] Moen, E. et al. Deep learning for cellular image analysis. *Nat. Methods* **16**, 1233–1246 (2019). 31133758 10.1038/s41592-019-0403-1 PMC8759575

[18] Ouyang, W., Aristov, A., Lelek, M., Hao, X. & Zimmer, C. Deep learning massively accelerates super-resolution localization microscopy. *Nat. Biotechnol.* **36**, 460–468 (2018). 29658943 10.1038/nbt.4106

[19] Nehme, E., Weiss, L. E., Michaeli, T. & Shechtman, Y. Deep-STORM: super-resolution single-molecule microscopy by deep learning. *Optica* **5**, 458–464 (2018).

[20] Lavoie-Cardinal, F. et al. Neuronal activity remodels the F-actin based submembrane lattice in dendrites but not axons of hippocampal neurons. *Sci. Rep.* **10**, 11960 (2020). 32686703 10.1038/s41598-020-68180-2 PMC7371643

[21] Bilodeau, A. et al. Microscopy analysis neural network to solve detection, enumeration and segmentation from image-level annotations. *Nat. Mach. Intell.* **4**, 455–466 (2022).

[22] Foroughi pour, A. et al. Deep learning features encode interpretable morphologies within histological images. *Sci. Rep.* **12**, 9428 (2022). 35676395 10.1038/s41598-022-13541-2 PMC9177767

[23] Wang, L. et al. An interpretable deep-learning architecture of capsule networks for identifying cell-type gene expression programs from single-cell RNA-sequencing data. *Nat. Mach. Intell.* **2**, 693–703 (2020).

[24] Alzubaidi, L. et al. Review of deep learning: concepts, CNN architectures, challenges, applications, future directions. *J. Big Data* **8**, 53 (2021). 33816053 10.1186/s40537-021-00444-8 PMC8010506

[25] Kohavi, R. A Study of cross-validation and bootstrap for accuracy estimation and model selection. In *14th International Joint Conference on Artificial Intelligence (IJCAI)* Vol. 2, 1137–1143 (Morgan Kaufmann Publishers Inc., 1995).

[26] Huang, G., Liu, Z., Van Der Maaten, L. & Weinberger, K. Q. Densely connected convolutional networks. In *Proc. IEEE Conference on Computer Vision and Pattern Recognition* 4700–4708 (IEEE, 2017).

[27] Xu, J. et al. Super-resolution imaging reveals the evolution of higher-order chromatin folding in early carcinogenesis. *Nat. Commun.* **11**, 1899 (2020). 32313005 10.1038/s41467-020-15718-7 PMC7171144

[28] Stevens, J. G., Wagner, E. K., Devi-Rao, G. B., Cook, M. L. & Feldman, L. T. RNA complementary to a herpesvirus alpha gene mRNA is prominent in latently infected neurons. *Science* **235**, 1056–1059 (1987). 2434993 10.1126/science.2434993

[29] Doll, J. R., Thompson, R. L. & Sawtell, N. M. Infectious herpes simplex virus in the brain stem is correlated with reactivation in the trigeminal ganglia. *J. Virol.* **93**, e02209–e02218 (2019). 30728262 10.1128/JVI.02209-18 PMC6450102

[30] Harkness, J. M., Kader, M. & DeLuca, N. A. Transcription of the herpes simplex virus 1 genome during productive and quiescent infection of neuronal and nonneuronal cells. *J. Virol.* **88**, 6847–6861 (2014). 24719411 10.1128/JVI.00516-14 PMC4054390

[31] Kokhlikyan, N. et al. Captum: a unified and generic model interpretability library for PyTorch. Preprint at https://arxiv.org/abs/2009.07896 (2020).

[32] Zeiler, M. D. & Fergus, R. Visualizing and understanding convolutional networks. In *Proc. 13th European Conference on Computer Vision* 818–833 (ECCV, 2014).

[33] Zhou, B., Khosla, A., Lapedriza, A., Oliva, A. & Torralba, A. Learning deep features for discriminative localization. In *IEEE Conference on Computer Vision and Pattern Recognition (CVPR)* 2921–2929 (IEEE, 2016).

[34] Karras, T. et al. Analyzing and improving the image quality of StyleGAN. In *Proc. IEEE/CVF Conference on Computer Vision and Pattern Recognition* 8110–8119 (IEEE, 2020).

[35] Abraham, K. J. et al. Nucleolar RNA polymerase II drives ribosome biogenesis. *Nature* **585**, 298–302 (2020). 32669707 10.1038/s41586-020-2497-0 PMC7486236

[36] Belthangady, C. & Royer, L. A. Applications, promises, and pitfalls of deep learning for fluorescence image reconstruction. *Nat. Methods* **16**, 1215–1225 (2019). 31285623 10.1038/s41592-019-0458-z

[37] Neguembor, M. V. et al. Transcription-mediated supercoiling regulates genome folding and loop formation. *Mol. Cell.* **81**, 3065–3081.e12 (2021). 34297911 10.1016/j.molcel.2021.06.009 PMC9482096

[38] Grosche, L. et al. Herpes simplex virus type 1 propagation, titration and single-step growth curves. *Bio. Protoc.* **9**, e3441 (2019). 10.21769/BioProtoc.3441 PMC7853997 33654936

[39] Bates, M., Huang, B., Dempsey, G. T. & Zhuang, X. Multicolor super-resolution imaging with photo-switchable fluorescent probes. *Science* **317**, 1749–1753 (2007). 17702910 10.1126/science.1146598 PMC2633025

[40] Martin, L. et al. A protocol to quantify chromatin compaction with confocal and super-resolution microscopy in cultured cells. *STAR Protoc.* **2**, 100865 (2021). 34632419 10.1016/j.xpro.2021.100865 PMC8488755

[41] Huang, B., Wang, W., Bates, M. & Zhuang, X. Three-dimensional super-resolution imaging by stochastic optical reconstruction microscopy. *Science* **319**, 810–813 (2008). 18174397 10.1126/science.1153529 PMC2633023

[42] Rust, M. J., Bates, M. & Zhuang, X. Sub-diffraction-limit imaging by stochastic optical reconstruction microscopy (STORM). *Nat. Methods* **3**, 793–795 (2006). 16896339 10.1038/nmeth929 PMC2700296

[43] Tokunaga, M., Imamoto, N. & Sakata-Sogawa, K. Highly inclined thin illumination enables clear single-molecule imaging in cells. *Nat. Methods* **5**, 159–161 (2008). 18176568 10.1038/nmeth1171

[44] Schindelin, J. et al. Fiji: an open-source platform for biological-image analysis. *Nat. Methods* **9**, 676–682 (2012). 22743772 10.1038/nmeth.2019 PMC3855844

[45] Pedregosa, F. et al. Scikit-learn: machine learning in Python. *J. Mach. Learn. Res.* **12**, 2825–2830 (2011).

[46] Buslaev, A. et al. Albumentations: fast and flexible image augmentations. *Information* **11**, 125 (2020).

[47] Chen, P., Liu, S., Zhao, H. & Jia, J. GridMask data augmentation. Preprint at https://arxiv.org/abs/2001.04086 (2020)

[48] Goutte, C. & Gaussier, E. A probabilistic interpretation of precision, recall and *F*-score, with implication for evaluation. *Lect. Notes Comput. Sci.* **3408**, 345–359 (2005).

[49] Hanley, J. A. & McNeil, B. J. The meaning and use of the area under a receiver operating characteristic (ROC) curve. *Radiology* **143**, 29–36 (1982). 7063747 10.1148/radiology.143.1.7063747

[50] Carnevali, D. et al. A deep learning method that identifies cellular heterogeneity using nanoscale nuclear features. *Code Ocean*10.24433/CO.7405455.v2 (2024). 10.1038/s42256-024-00883-x PMC11415298 39309215

