# Supplementary material for: A deep learning method that identifies cellular heterogeneity using nanoscale nuclear features
Source: Nat Mach Intell. 2024 Aug 27;6(9):1021–33. doi: 10.1038/s42256-024-00883-x (PMC11415298; doi:10.1038/s42256-024-00883-x)
Supplement: Supplementary file 1 — Supplementary Figs. 1 and 2. [file 42256_2024_883_MOESM1_ESM.pdf]

nature machine intelligence

Supplementary information https://doi.org/10.1038/s42256-024-00883-x

A deep learning method that identifies cellular heterogeneity using nanoscale nuclear features

In the format provided by the authors and unedited

Supplementary Material.

A deep learning method that identifies cellular heterogeneity using nanoscale nuclear features

Usage of GAN-generated images for nucleoli occlusion

To validate the importance of nucleoli for cell state discrimination, we modified the test set images by replacing the nucleoli regions with a random, non-nucleolus region taken from images generated by a Generative Adversarial Network (GAN) model.

During the GAN training process, authentic SR dual-color images were used to train a model capable of generating images indistinguishable from the real images by the naked eye (Supplementary Fig. 1a). 10x-rendered images from dual-color STORM localizations were uniformly resized to 1024×1024px. These images were used to train and validate a StyleGAN-2 ADA[1] GAN model using the Differentiable Augmentation method (DiffAugment)[2]. We applied the default ‘color, translation, cutout’ augmentation technique with *kimg* option set to 500. The purpose of this model was to generate thousands of synthetic images of human induced pluripotent stem cells (hiPSCs) and somatic cells, with 5000 images of each condition generated (as shown in Supplementary Fig. S1b). The quality of the simulated images was assessed using the common Fréchet Inception Distance (FID)[3] reaching high values of 40.7 for somatic and 37.5 for hiPSC cells.

The use of GAN-generated regions to occlude nucleoli in our images (Supplementary Fig. 2, a-b) significantly impacted the model's identification accuracy. The classification model accurately distinguishes between somatic cells and hiPSCs when tested on normal images. However, when nucleoli are occluded, the model misclassifies most images as somatic cells, indicating that these regions contain essential features for accurate classification. For hiPSCs, where nucleoli are key distinguishing features, accuracy notably decreased. Conversely, accuracy improved for somatic cells (Supplementary Fig. 2, c-f), where nucleoli are often considered distracting. These results highlight the importance of nucleoli in classifying hiPSCs and demonstrate the model's capability to identify these critical structures.

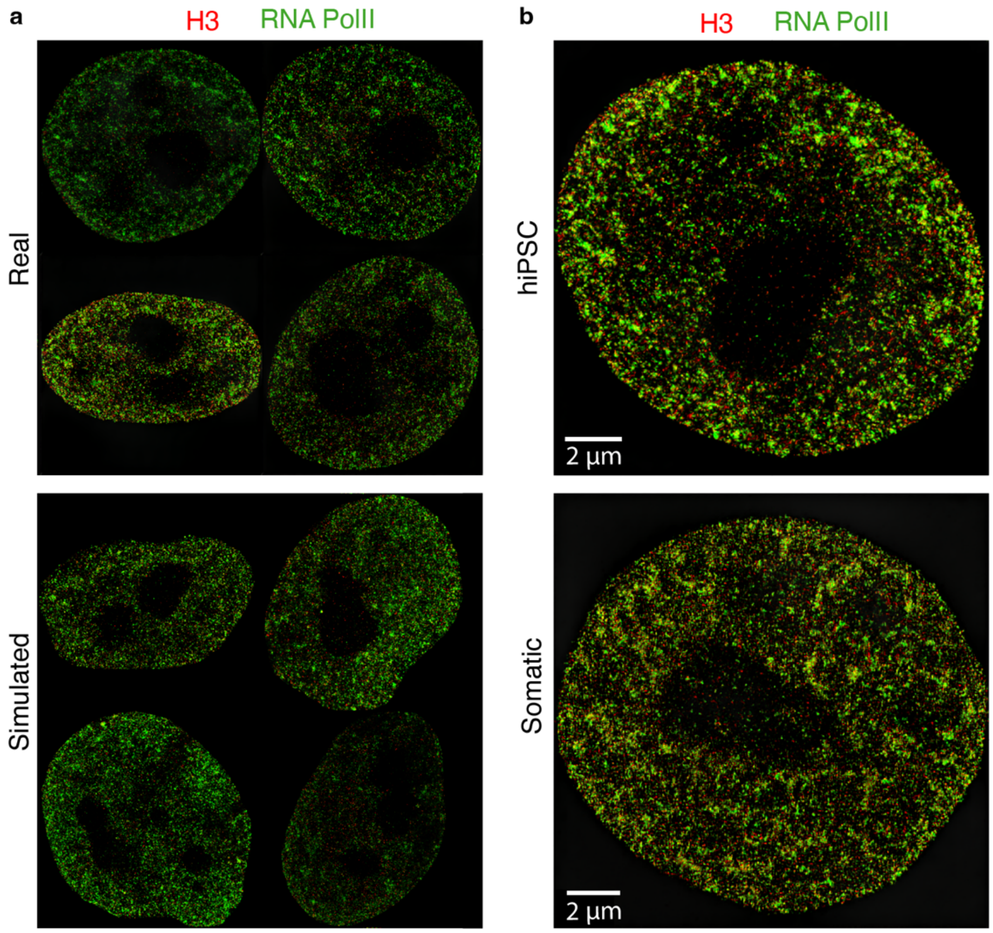

Supplementary Figure 1. (a) Image comparison between full-size 10x real images rendered from localization of Pol II and H3, and dual-color images simulated using GAN. (b) Representative dual-color Pol II and H3 simulated images generated by a trained GAN model using the DiffAugment method.

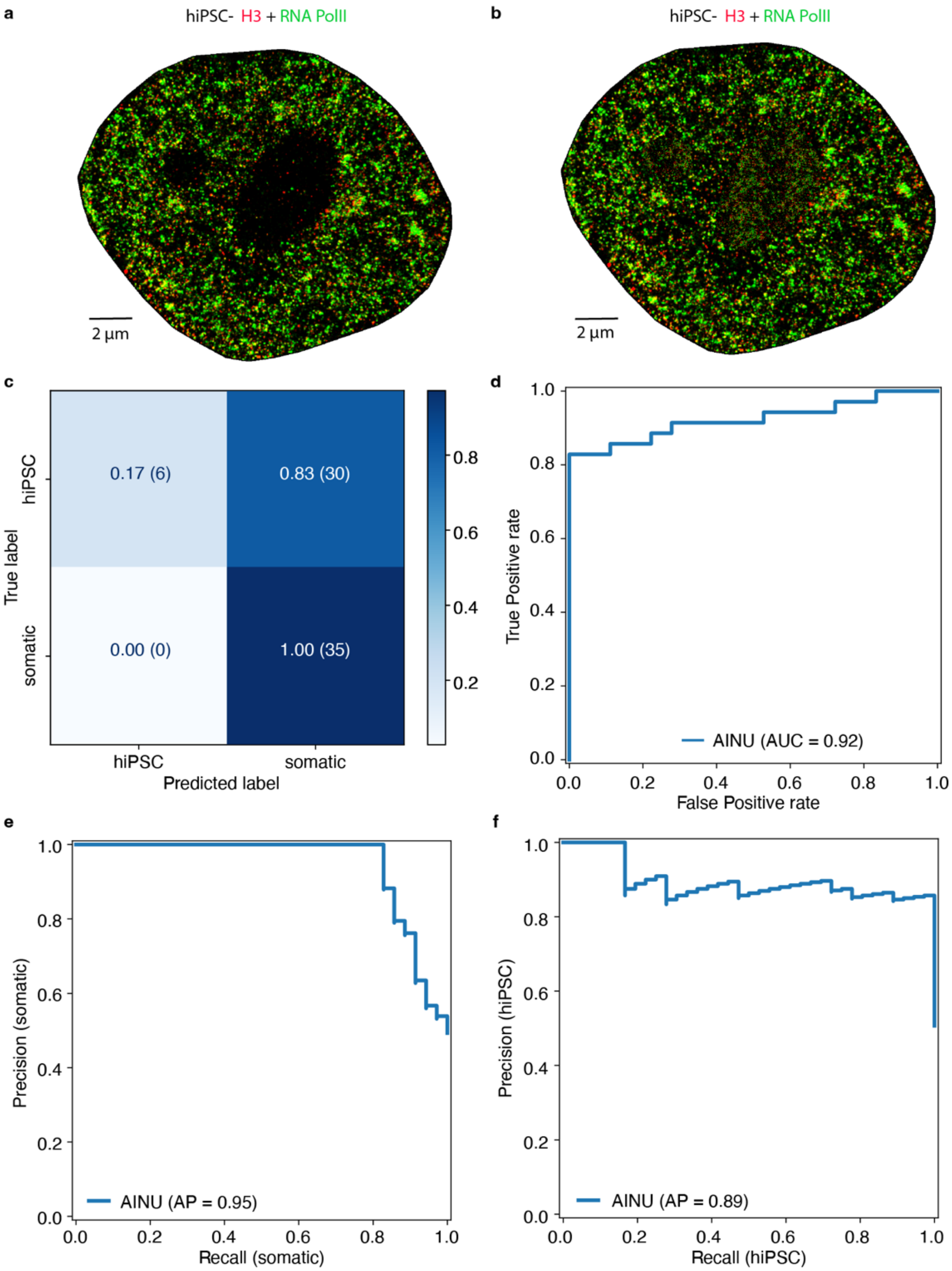

Supplementary Figure 2. AINU trained with Pol II and H3 images correctly identifies somatic cells and iPSCs. (a) Representative unmodified super-resolution image rendered at 10x magnification using dual-color Pol II and H3 localizations. (b) The same as in (a) with the nucleolus replaced with GAN-generated areas. (c,d) AINU trained with dual-color Pol II and H3 images was challenged on a test set of 71 previously unseen images with nucleoli occluded by filling them with GAN-generated areas. Normalized confusion matrix (with numbers of positively and negatively predicted images in parentheses). (c) shows the performance of the model for each class; the main diagonal reports the accuracy for each class. The ROC curve (d) shows the performance of the model at all classification thresholds and the value of the AUC. (e,f) Precision and recall plots for the somatic cell (e) and hiPSC (f) classes, reporting the overall average precision (AP).

Supplementary References

1. Lehtinen, J. & Aila NVIDIA, T. Analyzing and Improving the Image Quality of StyleGAN. In *Proceedings of the IEEE/CVF conference on computer vision and pattern recognition*, 8110–8119 (2016).
2. Zhao, S., Liu, Z., Lin, J., Zhu, J. Y. & Han, S. Differentiable Augmentation for Data-Efficient GAN Training. *Advances in Neural Information Processing Systems* **33,** 7559–7570 (2020).
3. Heusel, M., Ramsauer, H., Unterthiner, T., Nessler, B. & Hochreiter, S. GANs Trained by a Two Time-Scale Update Rule Converge to a Local Nash Equilibrium. *Advances in Neural Information Processing Systems* **30** (2017).
