# Supplementary material for: A deep learning method that identifies cellular heterogeneity using nanoscale nuclear features
Source: Nat Mach Intell. 2024 Aug 27;6(9):1021–33. doi: 10.1038/s42256-024-00883-x (PMC11415298; doi:10.1038/s42256-024-00883-x)
Supplement: Supplementary file 2 — Reporting Summary [file 42256_2024_883_MOESM2_ESM.pdf]

nature portfolio

Corresponding author(s): Maria Pia Cosma, Ignacio Arganda-Carreras

Last updated by author(s): Jun 12, 2024

Reporting Summary

Nature Portfolio wishes to improve the reproducibility of the work that we publish. This form provides structure for consistency and transparency in reporting. For further information on Nature Portfolio policies, see our Editorial Policies and the Editorial Policy Checklist.

Statistics

For all statistical analyses, confirm that the following items are present in the figure legend, table legend, main text, or Methods section.

n/a	Confirmed	
☐	☒	The exact sample size (*n*) for each experimental group/condition, given as a discrete number and unit of measurement
☐	☒	A statement on whether measurements were taken from distinct samples or whether the same sample was measured repeatedly
☐	☒	The statistical test(s) used AND whether they are one- or two-sided<br>*Only common tests should be described solely by name; describe more complex techniques in the Methods section.*
☒	☐	A description of all covariates tested
☒	☐	A description of any assumptions or corrections, such as tests of normality and adjustment for multiple comparisons
☐	☒	A full description of the statistical parameters including central tendency (e.g. means) or other basic estimates (e.g. regression coefficient) AND variation (e.g. standard deviation) or associated estimates of uncertainty (e.g. confidence intervals)
☐	☒	For null hypothesis testing, the test statistic (e.g. *F*, *t*, *r*) with confidence intervals, effect sizes, degrees of freedom and *P* value noted<br>*Give P values as exact values whenever suitable.*
☒	☐	For Bayesian analysis, information on the choice of priors and Markov chain Monte Carlo settings
☒	☐	For hierarchical and complex designs, identification of the appropriate level for tests and full reporting of outcomes
☒	☐	Estimates of effect sizes (e.g. Cohen's *d*, Pearson's *r*), indicating how they were calculated

Our web collection on statistics for biologists contains articles on many of the points above.

Software and code

Policy information about availability of computer code

Data collection	NIS element (4.60 and 5.21; Nikon) installed at N-STORM 4.0 microscope was used to acquire SR images of RNA PolII and H3.<br>ONI NimOS v.10.5 installed at Oxford Nanoimager microscope was used to extract DNA molecule localizations
Data analysis	Fiji (v. 2.9.0) software used for SR image analysis can be found at https://fiji.sc/<br>Insight3 (v. 4.29.8) software used for SR image processing has been kindly provided by Dr Bo Huang (UCSF)<br>R (v. 4.2.2) software for statistical analysis can be found at https://www.r-project.org/<br>GraphPad Prism (v. 8.01) software for statistical analysis can be found at https://www.graphpad.com/scientific-software/prism/<br>MatLab (v. 2016a) software used for data analysis can be found at https://www.mathworks.com/products/matlab.html<br>Python (v. 3.9.7) programming language used for data analysis can be found at https://www.python.org/<br>PyTorch (v. 1.10) framework used for DL model training/validation and testing can be found at https://pytorch.org/<br>All the codes used to process and analyze the data are available at https://doi.org/10.24433/CO.7405455.v2

For manuscripts utilizing custom algorithms or software that are central to the research but not yet described in published literature, software must be made available to editors and reviewers. We strongly encourage code deposition in a community repository (e.g. GitHub). See the Nature Portfolio guidelines for submitting code & software for further information.

Data

Policy information about availability of data

All manuscripts must include a data availability statement. This statement should provide the following information, where applicable:

- Accession codes, unique identifiers, or web links for publicly available datasets
- A description of any restrictions on data availability
- For clinical datasets or third party data, please ensure that the statement adheres to our policy

The trained model as well as the full dataset of dual-color images generated during the current study are available in the the Code Ocean capsule number 7405455 at https://doi.org/10.24433/CO.7405455.v2. For single-color images (Pol II and DNA) only the trained models and the test sets are available to reproduce the results of the paper.

Human research participants

Policy information about studies involving human research participants and Sex and Gender in Research.

Reporting on sex and gender	N/A
Population characteristics	N/A
Recruitment	N/A
Ethics oversight	N/A

Note that full information on the approval of the study protocol must also be provided in the manuscript.

Field-specific reporting

Please select the one below that is the best fit for your research. If you are not sure, read the appropriate sections before making your selection.

☒ Life sciences ☐ Behavioural & social sciences ☐ Ecological, evolutionary & environmental sciences

For a reference copy of the document with all sections, see nature.com/documents/nr-reporting-summary-flat.pdf

Life sciences study design

All studies must disclose on these points even when the disclosure is negative.

Sample size	CNN historically requires large amount of data to be trained while STORM Super-Resolution imaging is a low-throughput technique. Hence, being the first time SR images of cell nuclei are used to train a CNN for classification purpose, we started with a low number of sample images and increased the dataset until the model reached high performances.
Data exclusions	Data exclusion was performed only when acquisition defects were detected such as uneven illumination of the imaging field or suboptimal spatial drift correction. These are common practice quality check assessments in super-resolution imaging
Replication	Imaging data was collected In multiple imaging sessions performed in different days with independent samples (i.e. independent cell preparation/labelling); (ss)RT-qPCR experiment was performed in 3 biological replicates. Findings were replicated.
Randomization	Male and female human somatic and iPS cells were selected among 7 different cell types. Randomization was not relevant for HSV-1 infected cell.
Blinding	Since the different cell lines and conditions used for the study require different cell culture media and display different cell morphology, blinding cannot be applied during data collection.<br>For data analysis (i.e. training, validation and testing), sample images were randomly chosen by the DL algorithm with given proportion (60/20/20) for each cell type and condition

Reporting for specific materials, systems and methods

We require information from authors about some types of materials, experimental systems and methods used in many studies. Here, indicate whether each material, system or method listed is relevant to your study. If you are not sure if a list item applies to your research, read the appropriate section before selecting a response.

Materials & experimental systems

n/a	Involved in the study
☐	☒ Antibodies
☐	☒ Eukaryotic cell lines
☒	☐ Palaeontology and archaeology
☒	☐ Animals and other organisms
☒	☐ Clinical data
☒	☐ Dual use research of concern

Methods

n/a	Involved in the study
☒	☐ ChIP-seq
☒	☐ Flow cytometry
☒	☐ MRI-based neuroimaging

Antibodies

Antibodies used

Antibodies were used for cell imaging
Primary antibodies:
anti-RNA polymerase II CTD repeat YSPTSPS (phospho S5) antibody, Abcam, #ab5131; Histone H3 antibody, Active Motif, #39763.
Secondary antibodies:
donkey anti-rabbit IgG H&L (Alexa Fluor® 647), Abcam, #ab150075 for single-colour of RNA PolII;
For dual-colour imaging: home-made dye pair [Alexa Fluor™ 405 NHS Ester, Thermo Fisher,#A30000; Cy®3 Maleimide Mono-Reactive Dye Pack, Sigma-Aldrich, #GEPA23031; Alexa Fluor™ 647 NHS Ester (succinimidyl ester), ThermoScientific, #A20006] labeled secondary antibodies [AffiniPure donkey anti-rabbit IgG (H+L), Jackson ImmunoResearch, #711005152; Peroxidase AffiniPure goat anti-mouse IgG, Fcγ subclass 1 specific, Jackson ImmunoResearch, # 115-005-205;
For Extended Data Fig. 3g, anti-RNA polymerase II CTD repeat YSPTSPS (phospho S5; Abcam #ab5408) was used for STORM, and anti-NOLC1 (Abcam # ab184550) was used to mark nucleoli (both diluted 1:50). Secondary antibodies (goat anti-mouse IgG Alexa Fluor™ 647, ThermoScientific #A21235; and goat anti-rabbit IgG, Oregon Green 488, ThermoScientific #O-11038)

Validation

All primary antibodies are commercially available and suitable for Immunofluorescence applications. Their performance and specificity was validated by the producer company as declared in the Abcam website: https://www.abcam.com/en-es

Eukaryotic cell lines

Policy information about cell lines and Sex and Gender in Research

Cell line source(s)

A549 cells (lung carcinoma, American Type Culture Collection, ATCC CRL-185); B lymphocytes (GM12878); BJ fibroblasts (human foreskin skin fibroblasts) (American Type Culture Collection, ATCC CRL-2522); IMR90 (fibroblasts isolate from lung tissue, American Type Culture Collection, ATCC CCL-186); bone marrow mesenchymal stem cells (MSC, Guangzhou Salilai Stem cell Science and Technology Co.,LTD); HeLa; Müller glia; myocardiocytes (American Type Culture Collection, ATCC ACL16); spontaneously arising retinal pigment epithelium (ARPE-19) cells (China National Collection of Authenticated Cell Cultures, CSTR:19375.09.3101HUMGNHu45); urine epithelial cells (gift from Duanqing Pei, Guangzhou Institutes of Biomedicine and Health Chinese Academy, Guangzhou, China, https://doi.org/10.1038/nmeth.2283); Vero cells (African green monkey, Kidney epithelial, American Type Culture Collection, ATCC CCL-81); normal lung tissue fibroblast iPSC (hiPS(IMR90)-4: WiCell, #WISCi004); BJ fibroblast-iPSC (https://doi.org/10.1016/j.cell.2015.01.054); Amniocyte-iPSC (Shenzhen Cell Inspire Biotechnology Co.,Ltd, IPSN0008); Dermal fibroblast-iPSC (Shenzhen Cell Inspire Biotechnology Co.,Ltd, IPSN0010); Periosteum cell-iPSC (Shenzhen Cell Inspire Biotechnology Co.,Ltd, IPSN0011); Umbilical cord mesenchymal stem cell-iPSC (Shenzhen Cell Inspire Biotechnology Co.,Ltd, IPSN0039) and Urine epithelial cell-iPSC (https://doi.org/10.1186/s13287-021-02238-4 and https://doi.org/10.1038/srep22484)

Authentication

All the cell lines are commercially available or have been previously published and we did not perform any additional authentication procedure.

Mycoplasma contamination

All the cells were grown in BSL2 mycoplasma free culture room and regularly tested for mycoplasma. No contaminations were reported.

Commonly misidentified lines
(See ICLAC register)

No commonly misindentified cell lines were used in this study
